# Assisted Oocyte Activation With Calcium Ionophore Improves Pregnancy Outcomes and Offspring Safety in Infertile Patients: A Systematic Review and Meta-Analysis

**DOI:** 10.3389/fphys.2021.751905

**Published:** 2022-01-24

**Authors:** Yinghua Shan, Huishan Zhao, Dongmei Zhao, Jianhua Wang, Yuanqing Cui, Hongchu Bao

**Affiliations:** Reproductive Medicine Centre, The Affiliated Yantai Yuhuangding Hospital of Qingdao University, Yantai, China

**Keywords:** calcium ionophore, ICSI, miscarriage, congenital birth defects, pregnancy, assisted oocyte activation

## Abstract

This study aimed to evaluate the efficacy and safety of calcium ionophore during assisted oocyte activation (AOA). This meta-analysis contained randomized controlled trials and prospective observational and retrospective trials. The summary odds ratio (OR) with 95% confidence intervals (CIs) was calculated for clinical pregnancy rate and live birth rate. Both fixed and random effects models were applied. A total of 22 studies were included into this meta-analysis. Seventeen of the included studies showed that calcium ionophore increased the clinical pregnancy rate (OR, 2.14; 95% CI, 1.38–3.31). Similarly, 14 studies indicated that AOA with calcium ionophore during intracytoplasmic sperm injection (ICSI) improved the live birth rate considerably (OR, 2.65; 95% CI, 1.53–4.60). Moreover, fertilization, blastocyst formation, and implantation rate were higher after using AOA with calcium ionophore combined with ICSI. In addition, calcium ionophore did not increase top-quality embryo rate, cleavage rate, miscarriage rate, congenital birth defects, and neonatal sex ratio. Therefore, calcium ionophore followed by ICSI not only significantly improved live birth and overall pregnancy, but also did not affect the incidence of miscarriage, congenital birth defects, and neonatal sex ratio. This meta-analysis indicated that using calcium ionophore to activate oocytes was beneficial for couples with poor fertilization rates following ICSI.

## Introduction

*In vitro* fertilization (IVF) and intracytoplasmic sperm injection (ICSI) are common techniques in the field of assisted reproduction (Hamberger et al., 1998; Kissin et al., 2014). The number of ICSI cycles has increased notably as the number of infertile individuals has increased, with average fertilization rates estimated to reach 70%, but fertilization failure still occurs in 1–5% of ICSI cycles (Bhattacharya et al., 2013; Johnson et al., 2013; ESHRE/Alpha, 2017). ICSI enables male factor infertility to be treated in fertilization. The failure of standard ICSI to fertilize patients with a sufficient number of oocytes (usually >3) is primarily due to oocyte activation failure, which may be interrelated with oocyte or sperm factors (Vanden Meerschaut et al., 2014; Bonte et al., 2019).

Oocyte activation is a spatial-temporal, regulated process, and caused by a series of intracellular Ca^2+^ oscillations from sperm entry into the ooplasm during sperm-oocyte fusion (Stricker, 1999; Swain and Pool, 2008). This process is triggered by sperm-borne phospholipase C zeta (PLC zeta) which is transported to the oocyte at sperm entry into the ooplasm (Saunders et al., 2002). Ca^2+^ oscillations are recognized as indispensable for successful oocyte activation, fertilization, and embryogenesis (Ramadan et al., 2012). Insufficient calcium may enable fertilization failure and cleavage anomalies. Thus, increasing appropriate calcium artificially during the ICSI period could avoid the above situation.

Currently, assisted oocyte activation (AOA) in conjunction with ICSI has been developed and broadly applied in improving clinical outcomes of male factor infertility, embryo developmental block, and patients who remain infertile after ICSI (Borges et al., 2009a; Kashir et al., 2010; Montag et al., 2012; Kang et al., 2015; Ferrer-Buitrago et al., 2018). AOA methods include electrical, mechanical, and chemical activation, of which calcium ionophore belonging to chemical AOA is the most universally used and most effective technique, and it activates oocytes not only by enhancing intracellular Ca^2+^ but also with the same recruiting method from the culture medium outside the oocyte (Kashir et al., 2010; Amdani et al., 2013; Nomikos et al., 2013; Vanden Meerschaut et al., 2014; Yeste et al., 2016). Calcimycin (Yoon et al., 2013; Ebner et al., 2014) and ionomycin (Deemeh et al., 2014) are two commonly used calcium ionophores. Calcimycin (also referred to as A23187) can be either home-made or a ready-to-use solution called GM508 CultActive (Gynemed).

Recently, calcium ionophore has been widely used in various centers and obtained satisfactory results. Yet, considering that calcium oscillation is consistent with the active and rapid demethylation of the paternal genome and passive DNA demethylation of the maternal genome, it appears reasonable that an altered calcium signal, as reported following calcium ionophore treatment, might be related to modifications in genomic imprinting (Nikiforaki et al., 2016). Moreover, several meta-analysis studies assessed the value of calcium ionophore during AOA in improving reproductive outcomes, and researchers found that ICSI-AOA played a positive role in enhancing fertilization rate and clinical pregnancy outcome (Sfontouris et al., 2015; Murugesu et al., 2017). However, the small number of research samples reduces the credibility of AOA effectiveness. Identifying and evaluating more cases would contribute to more reliable conclusions.

The effect of ICSI-AOA on post-implantation embryo development and pregnancy effectiveness must be taken into consideration deeply. With the widespread application of chemical activators, their safety in offspring has attracted more and more attention. In this meta-analysis, we aim to identify, appraise, and assess the efficacy and safety of calcium ionophore as a method of AOA in promoting pregnancy outcomes and reducing miscarriage and congenital birth defects.

## Materials and Methods

This meta-analysis and systematic review was conducted and reported according to PRISMA guidelines.

### Search Strategy

PubMed, Embase, Scopus, and Cochrane Library databases were searched up to February 2020. The language and publication date were not limited. The following Medical Subject Headings (MeSH) terms and free-text terms were employed in searching the databases. MeSH terms included infertility and calcium ionophores. Free-text terms consisted of calcium ionophore, ionophores, calcium, A23187, calcimycin, ionmycin, AOA, oocyte activation, eggs activation, artificial eggs activation, assisted eggs activation, sterility reproductive, sterility, reproductive, subfertility, sub-fertility, infertility, ICSI, IVF, embryo transfer, blastocyst, miscarriage, pregnancy, and birth and congenital birth defects. The terms were combined with “AND” or “OR” Boolean operators. We expanded the search by using the “related article” function. Furthermore, we manually searched the reference list to identify any articles not captured by electronic database searches. Any search filters or limitations were not used in this study. In addition, full-text articles were authorized for inclusion. Using the aforementioned strategies, two groups of ICSI-AOA with calcium ionophore and ICSI-only (non-AOA) were compared and identified, and related data were extracted.

### Eligibility Criteria

All randomized controlled trials (RCTs) and observational studies involving comparison between “ICSI-AOA with calcium ionophore” with “ICSI-only (non-AOA)” patient groups and also those containing outcome indicators were included in this study. We excluded non-controlled studies that only reported outcome incidence and did not report outcome indicators.

### Data Extraction

Data collection of each study was completed by two independent reviewers (YS and HZ). Then a standardized Excel spreadsheet was used for performing quantitative data entry and processing. The accuracy of the collected and extracted data was acknowledged by a third reviewer (DZ). The content of data extraction was as follows: characteristics (author, publication year, country, study type, study period, study population, and oocyte activation); study groups (ICSI-AOA infertility patients with calcium ionophore vs. ICSI-only (non-AOA) control group); and outcome indicators (fertilization, cleavage, top-quality embryo, blastocyst, implantation, miscarriage, clinical pregnancy, live births, congenital birth defects, and neonatal sex ratio). The detailed information is summarized in Table 1. When multiple publications were involved in the same patient cohort, we chose the largest sample study.

**Table 1:** Study characteristics.

**No**.	**References**	**Country**	**Study type**	**Study period**	**Study population**	**Oocyte activation protocol**	**Control**	**Cases number**	**Outcome indicators**
1	Li J. et al. (2019)	China	Sibling oocytes control study-prospective cohort study	Oct 2015–Dec 2017	Previous ICSI cycles of no or low fertilization or were diagnosed with severe teratozoospermia	Calcium ionophore (ionomycin) exposure twice for 5 min with a 30 min interval	Sibling oocytes	50 patients Survival MII oocytes after ICSI: 513 oocytes AOA: 262 oocytes; control: 251 oocytes	Fertilization rate, Cleavage rate, blastocyst rate, Implantation rate, Pregnancy rate, Live-birth rate, Abortion rate, Fetal defect
2	Bonte et al. (2019)	Belgium	Retrospective cohort study	Apr 2001–Apr 2018	Previous ICSI cycles of no or low fertilization (≤33.3%)	CaCl_2_ injection and Calcium ionophore (ionomycin) exposure twice for 10 min with a 30 min interval	Before-after self contrast	122 couples AOA: 191 cycles, 2,476 oocytes; control: 243 cycles, 2351 oocytes	Fertilization rate, Pregnancy rate, Live-birth rate, Abortion rate, Fetal defect
3	Li B. et al. (2019)	China	Retrospective cohort study	Jan 2011–Dec 2016	(1) ICSI fertilization rate ≤50; (2) good quality embryo rate ≤30%; (3) severe oligoasthenoteratozoospermia (OAT); (4) TESA or PESA	Calcium ionophore (ionomycin) exposure for 10 min	Conventional ICSI	AOA:169 patients control:507 patients	Implantation rate, Pregnancy rate, Live-birth rate, Abortion rate, Fetal defect
4	Fawzy et al. (2018)	Egypt	RCT	Apr 2015–Jan 2016	Two previous ICSI cycles of low fertilization (0–30%) or with male-factor infertility undergoing their first ICSI cycle	Group I (SrCl_2_ for 60 min post-ICSI) group II (GM508 CultActive for 20 min post-ICSI)	Randomized controlled	343 participants (SrCl2 AOA:115, calcimycinAOA:113 and ICSI:115)	Fertilization rate, Cleavage rate, top Embryo rate, Blastocyst rate, Implantation rate, Pregnancy rate, Live-birth rate, Abortion rate
5	Mateizel et al. (2018)	Belgium	Single-center observational study- prospective cohort study	2004–2015	Low (*n* = 14) or no (*n* = 16) fertilization in previous cycles, globozoospermia (*n* = 6), small acrosome (*n* = 2), and poor embryo development (*n* = 1)	CaCl_2_ injection and Calcium ionophore (ionomycin) exposure twice for 7 min with a 30 min interval or calcium ionophore (GM508 CultActive) exposure for 15 min	Conventional ICSI	237 cycles, 74 pregnancies with ICSI+AOA, 47 newborns.	Fetal defect
6	Miller et al. (2016)	Israel	Retrospective cohort study	2006–2014	Failed fertilization after one ICSI procedure in the presence of at least five mature oocytes without oocyte abnormality or had <10% fertilization rate	Calcium ionophore (A23187) exposure for 10 min	Conventional ICSI	AOA: 83 pregnancies; ICSI: 595 pregnancies.	Abortion rate, fetal defect
7	Aydinuraz et al. (2016)	Turkey	Prospective, randomized and single blind study	Dec 2013–Feb 2014	Teratozoospermia (<4% according to WHO 2010 reference limits) and a low fertilization rate (<50%) in the previous cycle	Calcium ionophore (GM508 CultActive) exposure for 15 min	Sibling oocytes	21 couples AOA: 97 MII oocytes; Control: 97 MII oocytes	Fertilization rate, cleavage rate, top embryo rate, implantation rate, pregnancy rate, live-birth rate, abortion rate
8	Dayong Hao et al. (2016)	China	Sibling oocytes control study	Jan 2015–Dec 2015	Previous ICSI cycles of low fertilization ≤30%, globozoospermia, or poor embryo development	Calcium ionophore (A23187) exposure for 15 min	Sibling oocytes	AOA: 12 patients; ICSI: 12 patients	Fertilization rate, top embryo rate, fetal defect
9	Ebner et al. (2015)	Germany/ Austria	Prospective multicentre study	Duration almost 2-year	Previous ICSI cycles of low fertilization <50%, at least three cumulus–oocyte complexes	Calcium ionophore (GM508 CultActive) exposure for 15 min	Before-after self contrast	AOA: 101 cycles; Control: 101 cycles	Fertilization rate, blastocyst rate
10	Caglar Aytac et al. (2015)	Turkey	Prospective, randomized controlled study	Jan 2014–Aug 2014	DOR and partners with normal sperm parameters	Calcium ionophore (GM508 CultActive) exposure for 15 min	Randomized controlled	AOA: 148 patients; Control: 148 patients	Fertilization rate, cleavage rate, pregnancy rate, live-birth rate
11	Kang et al. (2015)	Korea	Retrospective study	Jan 2006–Jun 2013	Previous ICSI cycles of no or low fertilization (<45%)	Calcium ionophore (A23187) exposure for 5 min	Conventional TESE	AOA: 29 cycles; Control: 480 cycles	Pregnancy rate, live-birth rate
12	Ebner et al. (2014)	Austrian	Prospective, multicenter, uncontrolled intervention study	Duration 1 year	Complete developmental arrest (no transfer), or complete developmental delay (no morula/blastocyst on Day 5), or significantly reduced blastocyst formation (≤15%) in a previous cycle	Calcium ionophore (GM508 CultActive) exposure for 15 min	Before-after self contrast	AOA: 57 cycles; Control: 57 cycles	Fertilization rate, cleavage rate, top embryo rate, blastocyst rate, implantation rate, pregnancy rate, live-birth rate, abortion rate, fetal defect
13	Deemeh et al. (2014)	Iran	Historical cohort study	Feb 2008–May 2010	(1) Testicular sperm extraction (TESE); (2) severe teratozoospermia (sperm with abnormal morphology ≥98%)	Calcium ionophore (ionomycin) exposure for 10 min	Conventional ICSI	AOA-ICSI: 275 cycle (TESE: 150, severe teratozoospermia: 125); Control: 406 cycle	Pregnancy rate, live-birth rate, abortion rate, fetal defect
14	Yoon et al. (2013)	America	Retrospective study	2007–2011	Previous ICSI cycles of no or low fertilization (≤50%)	Calcium ionophore (A23187) exposure for 30 min	Before-after self contrast	AOA: 185 cycles; Control: 185 cycles	Implantation rate, pregnancy rate, live-birth rate, abortion rate, fetal defect
15	Eftekhar et al. (2013)	Iran	Prospective, randomized, unblinded, clinical trial	Apr 2012–Dec 2012	Teratoospermic partner (normal morphology <14%) undergoing to ICSI cycles	Calcium ionophore(A23187) exposure for 5 min	Randomized controlled	AOA: 19 patients; ICSI:19 patients	Pregnancy rate, live-birth rate, abortion rate
16	Ebner et al. (2012)	Germany/ Austria	Prospective multicenter study	Aug 2009–Mar 2011	Patients with azoospermia (44%) or cryptozoospermia (56%) (e.g., showing <0.1 spermatozoa/mL)	Calcium ionophore (GM508 CultActive) exposure for 15 min	Before-after self contrast	AOA: 75 cycles; Control: 88 cycles	Fertilization rate, blastocyst rate, implantation rate, pregnancy rate, live-birth rate, fetal defect
17	Montag et al. (2012)	Germany	Retrospective cohort study	2003–2009	Patients with 0, 1–29, or 30–50% fertilization in a previous ICSI cycle	Calcium ionophore (A23187) exposure for 15 min	Before-after self contrast	AOA: 129 cycles; Control: 103 cycles	Fertilization rate, implantation rate, pregnancy rate, live-birth rate, abortion rate, fetal defect
18	Vanden Meerschaut et al. (2012)	Belgium	Prospective case series	Jan 2006–Dec 2011	Previous ICSI cycles of no or low fertilization in whom the MOAT >84%	CaCl_2_ injection and Calcium ionophore (ionomycin) exposure twice for 10 min with a 30 min interval	Sibling oocytes	AOA: 14patients Control: 14 patients	Fertilization rate
19	Borges et al. (2009a)	Brazil	RCT	Jan 2006–Jul 2007	(1) Testicular sperm aspiration in non-obstructive-azoospermic patients (TESA-NOA group, *n* = 58); (2) TESA in obstructive- azoospermic patients (TESA-OA group, *n* = 48); (3) and percutaneous epididymal sperm aspiration in obstructive-azoospermic patients (PESA-OA, *n* = 98)	Calcium ionophore (A23187) exposure for 30 min	Randomized controlled	TESA-NOA AOA: 29 cycles; Control: 29 cycles; TESA-OA control: 24 cycles; AOA: 24 cycles; PESA-OA control: 49 cycles; AOA: 49cycles	Fertilization rate, top embryo rate, implantation rate, pregnancy rate
20	Borges et al. (2009b)	Brazil	RCT	Jan 2006–Jul 2007	The ejaculated group (*n* = 92), the epididymal group (*n* = 82), and the testicular group (*n* = 140)	Calcium ionophore (A23187) exposure for 30 min	Randomized controlled	Ejaculated AOA: 46 cycles; Control: 46 cycles Epididymal; AOA: 41 cycles; Control: 41 cycles; Testicular AOA: 70 cycles; Control:70 cycles	Fertilization rate, top embryo rate, implantation rate, pregnancy rate, abortion rate
21	Heindryckx et al. (2008)	Belgium	Prospective cohort study	None	Failed or low fertilization in previous ICSI cycles or who had well-known sperm-borne activation deficiencies such as globozoospermia	CaCl_2_ injection and Calcium ionophore (ionomycin) exposure twice for 10 min with a 30 min interval	Before-after self contrast	AOA: 30 patients; Control: 30 patients	Fertilization rate, pregnancy rate, live-birth rate, fetal defect
22	Kyono et al. (2012)	Japan	Retrospective study	1 April 2004 and 31 October 2010	Previous ICSI cycles of low fertilization (≤30%)	Group I (SrCl2 for 60–120 min post-ICSI) group II (A23187 5–10 min post-ICSI)	Fetal defect: conventional ICSI Fertilization rate/Pregnancy rate/Implantation rate/abortion rate: before-after self contrast	SrCl2: 35 patients A23187: 50 patients; Conventional ICSI: 530 patients	Fertilization rate, pregnancy rate, implantation rate, abortion rate, fetal defect

### Risk of Bias and Quality Assessment

Five RCTs and 17 observational studies that evaluated the association between AOA with calcium ionophore and clinical outcome were included in this study. The quality assessment of RCTs was performed through the Cochrane risk of bias assessment tool (Higgins, 2011). Seven domains were considered in the risk of bias assessment: random sequence generation (selection bias), allocation concealment (selection bias), blinding of participants and personnel (performance bias), blinding of outcome assessment (detection bias), incomplete outcome data (attrition bias), selective reporting (reporting bias), and other bias. The risk evaluation result was categorized as “low risk (with less than two high-risk components),” “unclear risk (with more than three unclear-risk components),” or “high risk (with more than four high-risk components)” of bias.

The risk of observational studies was evaluated using the Newcastle-Ottawa Quality Assessment Scale (NOS), which used a score system based on three categories: selection of subjects, comparability of study groups, and the assessment of exposure. According to the NOS score, we graded the studies as low quality (0–3 points), medium quality (4–6 points), and high quality (7–9 points). The maximum score is 9 points. The above quality assessment was undertaken by two reviewers (JW and YC) independently, and further confirmed by a third reviewer (YS). A funnel plot was used to assess publication bias.

### Outcomes of Interest

The main purpose of this meta-analysis was to appraise the efficacy and safety of AOA with calcium ionophore in combination with ICSI compared with the regular ICSI process, including clinical pregnancy rate and live birth rate. The secondary outcomes of interest were: fertilization rate, cleavage rate, top-quality embryo rate, blastocyst formation rate, implantation rate, miscarriage rate, congenital birth defects, and neonatal sex ratio. The explanation of each noun is shown as follows (Zegers-Hochschild et al., 2009; ESHRE/Alpha, 2017): Clinical pregnancy means a pregnancy diagnosed by ultrasonographic visualization of one or more gestational sacs to define clinical signs of pregnancy. Clinical pregnancy rate is the number of clinical pregnancies expressed per 100 embryo transfer cycles. Fertilization rate is 2PN zygotes divided by the number of injected mature oocytes. Cleavage rate is defined as the proportion of zygotes which cleave into embryos on day 2 post-insemination. Top-quality embryo rate is defined as the proportion of day 3 embryos with a high score or grade. Blastocyst formation rate is defined as the proportion of 2PN zygotes which are at the blastocyst stage at day 5 or day 6. Implantation rate is the number of gestational sacs observed divided by the number of embryos transferred. Live birth rate is defined as the number of deliveries that resulted in at least one live born baby, expressed per 100 embryo transfer cycles. Congenital birth defects refer to all structural, functional, and genetic anomalies diagnosed in the neonatal period.

### Statistical Analysis

A direct comparison meta-analysis was reported via odds ratio (OR) with 95% confidence intervals (CIs). We measured pooled ORs through a fixed effects model and calculated heterogeneity by *Q*-test and *I*^2^ statistics. A random effects model was employed instead when significant heterogeneity appeared with *P* < 0.1 and *I*^2>^ 50%. Both fixed and random effects models were applied in this study. We conducted subgroup analyses based on clinical diversity and performed two subgroup analyses. Subgroup analyses were stratified into two categories: “previous fertilization failure or low fertilization rate” and “embryo developmental problems [embryonic development block or sperm factor or diminished ovarian reserve (DOR)].” All statistical analysis was accomplished using Review Manager Version 5.3 statistical software and STATA 13.0.

## Results

### Study Selection and Characteristics

**Figure 1:**
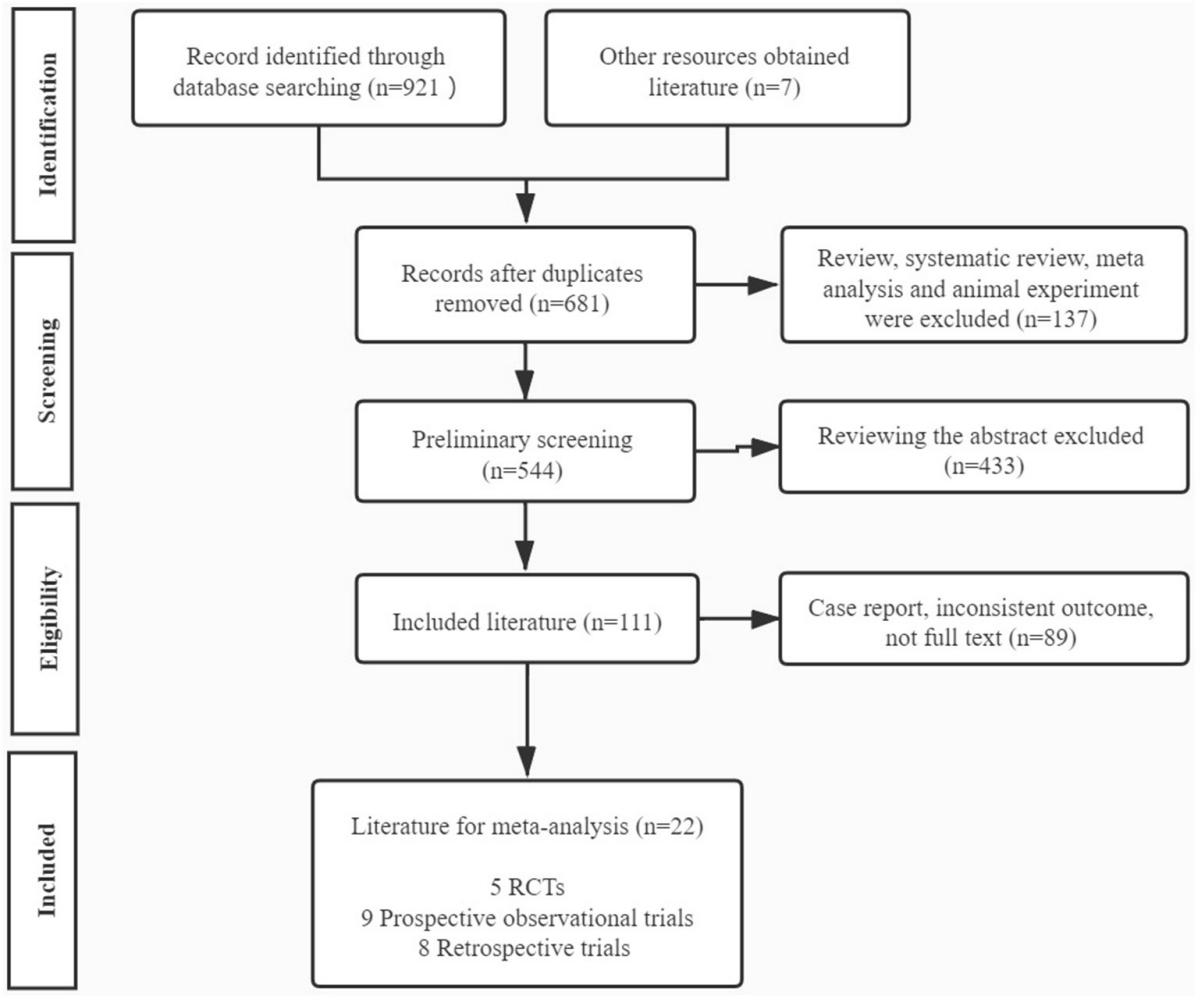
Flowchart of the search method and selection.

Our study initially yielded 921 articles via the database search and 7 articles through other resources, of which 681 studies were reserved after removing the duplicates. Then 137 articles including reviews, meta-analysis, and animal experiments were excluded. A total of 544 articles were initially screened out, and 433 articles were excluded by reading abstracts. Subsequently, 111 papers conformed to our eligibility criteria, of which 89 studies were precluded for the following reasons: case report, inconsistent outcome, or no full text. Eventually, 22 full-text articles were included in our meta-analysis, consisting of 5 RCTs (Borges et al., 2009a,b; Eftekhar et al., 2013; Caglar Aytac et al., 2015; Fawzy et al., 2018), 9 prospective observational trials (Heindryckx et al., 2008; Ebner et al., 2012, 2014, 2015; Vanden Meerschaut et al., 2012; Aydinuraz et al., 2016; Dayong Hao et al., 2016; Mateizel et al., 2018; Li J. et al., 2019), and 8 retrospective trials (Kyono et al., 2012; Montag et al., 2012; Yoon et al., 2013; Deemeh et al., 2014; Kang et al., 2015; Miller et al., 2016; Bonte et al., 2019; Li B. et al., 2019; Table 1). The flowchart of literature identification and selection is summarized in Figure 1.

### Methodological Quality Assessment

The quality of these five RCTs was evaluated using the Cochrane Risk of Bias tool. Only one study was considered to have high quality, and the remaining four articles all reached a moderate risk of bias (Supplementary Figure 1). The RCTs were regularly graded as high risk with complete blinding. To assess the quality of the included observational studies, the NOS was adopted to divide the studies into good, fair, and poor. The great majority of articles indicated good quality at a low risk of bias (six studies scored 9 points, three studies scored 8 points, and five studies scored 7 points), and the other three studies were recognized as medium quality at moderate risk (two studies scored 6 points and one study scored 5 points). The overall quality of the observational studies was high (Supplementary Table 1). Sensitivity analyses and publication bias assessments were executed for remarkable heterogeneity among studies.

### Primary Outcome

#### Pregnancy Rate

**Figure 2:**
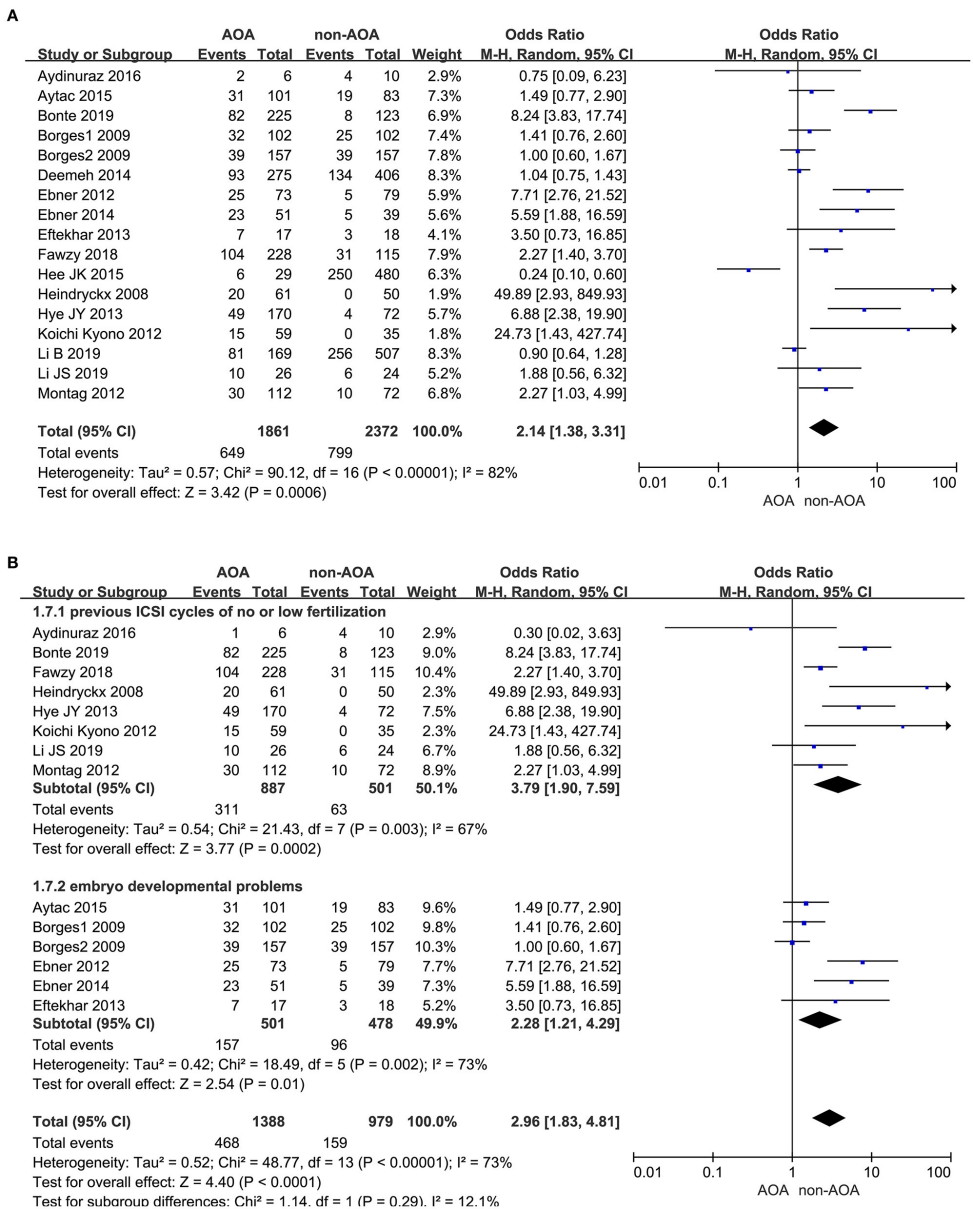
**(A)** Forest plot of pregnancy rate between AOA treatment and non-AOA treatment patient groups using calcium ionophore. **(B)** The subgroup analysis of pregnancy rate between AOA treatment and non-AOA treatment patient groups.

In this analysis, 17 studies investigated the effect of AOA with calcium ionophore on the pregnancy rate especially (Heindryckx et al., 2008; Borges et al., 2009a,b; Ebner et al., 2012, 2014; Kyono et al., 2012; Montag et al., 2012; Eftekhar et al., 2013; Yoon et al., 2013; Deemeh et al., 2014; Caglar Aytac et al., 2015; Kang et al., 2015; Aydinuraz et al., 2016; Fawzy et al., 2018; Bonte et al., 2019; Li B. et al., 2019; Li J. et al., 2019). It was 34.87% (649/1,861) in the ICSI-AOA with calcium ionophore group and 33.68% (799/2,372) in the non-AOA group. Patients undergoing AOA with calcium ionophore had a higher pregnancy rate compared with the control (OR 2.14; 95% CI, 1.38–3.31; *P* = 0.0006; *I*^2^= 82%; Figure 2A). The funnel plot of pregnancy rate was not asymmetric and Egger's test showed no statistically significance (*P* = 0.064), which suggested no publication bias (Supplementary Figure 2A). Considering the heterogeneity was statistically significant (*P* < 0.00001; *I*^2^= 82%), we conducted subgroup analyses. In the “previous fertilization failure or low fertilization rate” subgroup (OR 3.79; 95% CI, 1.90–7.59; *P* = 0.0002; *I*^2^= 67%) and “embryo developmental problems (embryonic development block or sperm factor or DOR)” subgroup (OR 2.28; 95% CI, 1.21–4.29; *P* = 0.01; *I*^2^= 73%), the results illustrated that AOA with calcium ionophore uncommonly enhanced the pregnancy rate (Figure 2B) in both subgroups, which indicated that the included study populations were not the main sources of variance in the pregnancy rate. The funnel plot showed no publication bias (Supplementary Figure 2B).

#### Live Birth Rate

**Figure 3:**
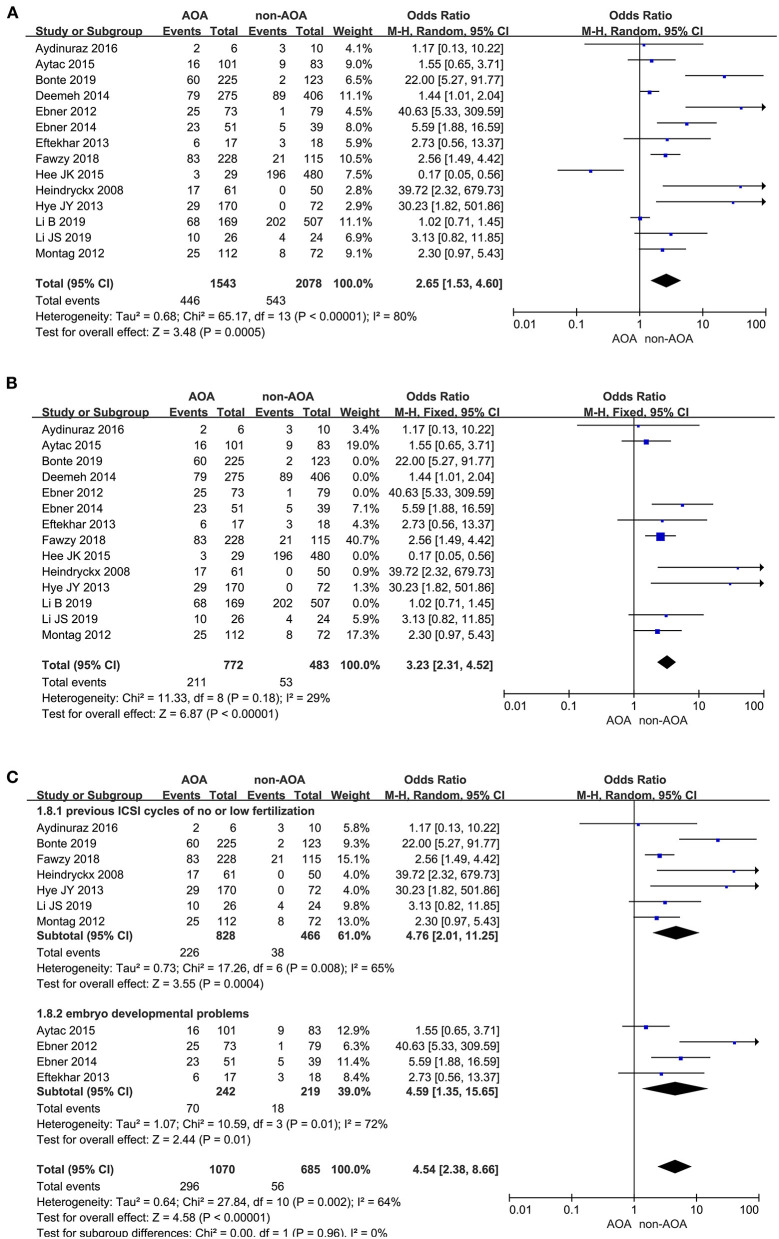
**(A)** Forest plot of live birth rate between AOA treatment and non-AOA treatment patient groups using calcium ionophore. **(B)** Forest plot of live birth rate between AOA treatment and non-AOA treatment patient groups after reducing the heterogeneity. **(C)** The subgroup analysis of live birth rate between AOA treatment and non-AOA treatment patient groups.

A total of 14 studies reported the live birth rate (Heindryckx et al., 2008; Ebner et al., 2012, 2015; Montag et al., 2012; Eftekhar et al., 2013; Yoon et al., 2013; Deemeh et al., 2014; Caglar Aytac et al., 2015; Kang et al., 2015; Aydinuraz et al., 2016; Fawzy et al., 2018; Bonte et al., 2019; Li B. et al., 2019; Li J. et al., 2019). The analysis showed that AOA with calcium ionophore heightened the offspring live birth rate. The funnel plots showed obvious publication bias among these articles (Supplementary Figure 3A). Owing to the *I*^2^ value (80%) representing high heterogeneity among the included studies, the pooled OR was performed through a random effects model (OR 2.65; 95% CI, 1.53–4.60; *P* = 0.0005; *I*^2^= 80%; Figure 3A), and further subgroup analyses were executed. Moreover, the heterogeneity reduced (OR 3.23; 95% CI, 2.31–4.52; *P* < 0.00001; *I*^2^= 29%) and publication bias disappeared when we excluded the five low-quality studies (Ebner et al., 2012; Kang et al., 2015; Mateizel et al., 2018; Bonte et al., 2019; Li B. et al., 2019; Figure 3B), and the results were consistent with before the elimination. In subgroup analyses, consistent results revealed that AOA with calcium ionophore significantly increased the birth rate of re-assisted pregnancy in the “previous fertilization failure or low fertilization rate” subgroup (OR 4.76; 95% CI, 2.01–11.25; *P* = 0.0004; *I*^2^= 65%) and “embryo developmental problems (embryonic development block or sperm factor or DOR)” subgroup (OR 4.59; 95% CI, 1.35–15.65; *P* = 0.01; *I*^2^= 72%; Figure 3C), and no publication bias appeared (Supplementary Figure 3B). Hence, the high heterogeneity in this part was caused by the design and quality of studies but not the fact that included studies recruited diverse study populations.

### Secondary Outcomes

#### Fertilization Rate

**Figure 4:**
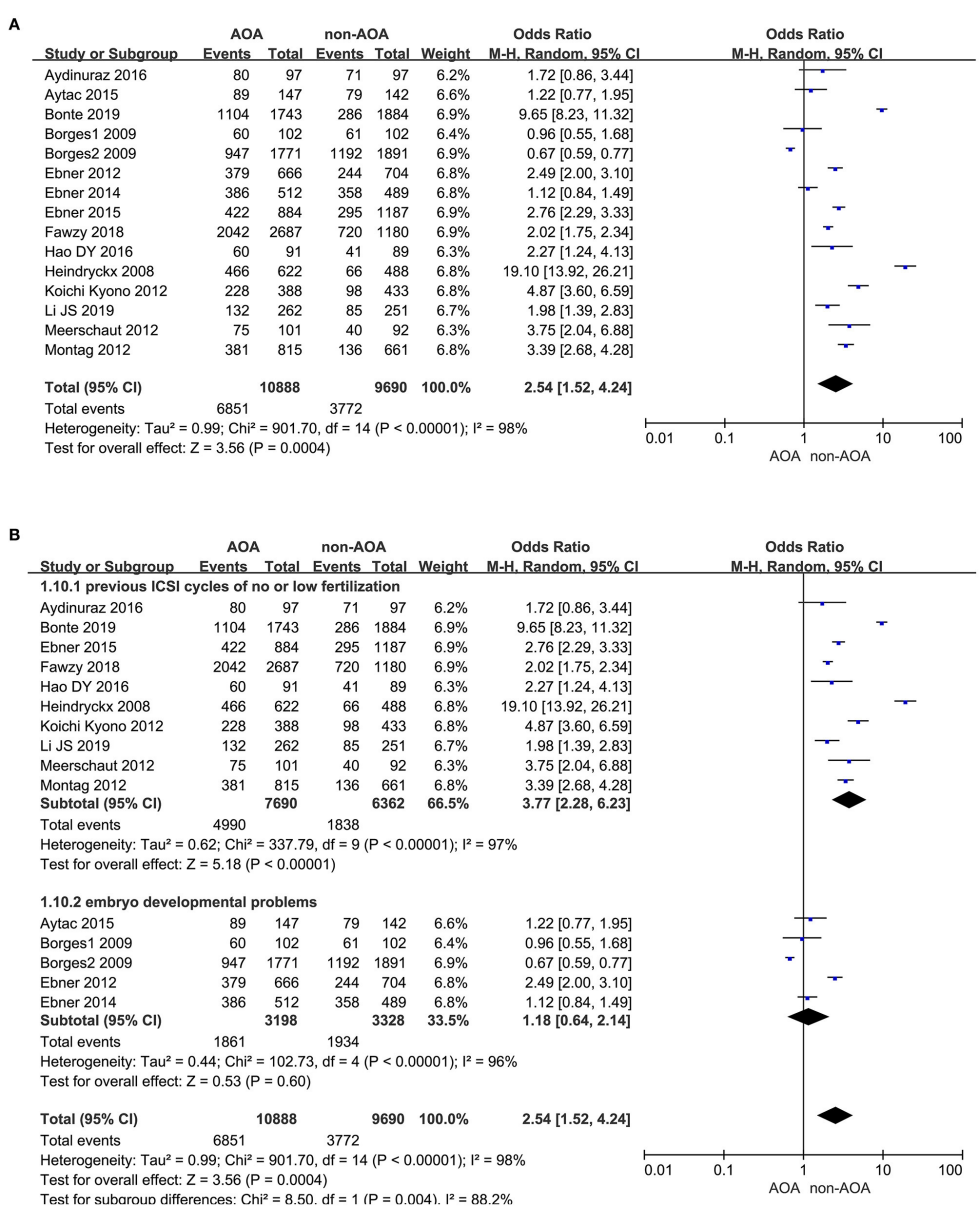
**(A)** Forest plot of fertilization rate between AOA treatment and non-AOA treatment patient groups using calcium ionophore. **(B)** The subgroup analysis of fertilization rate between AOA treatment and non-AOA treatment patient groups.

Fifteen articles investigated the effect of AOA with calcium ionophore on fertilization rate (Heindryckx et al., 2008; Borges et al., 2009a,b; Ebner et al., 2012, 2014, 2015; Kyono et al., 2012; Montag et al., 2012; Vanden Meerschaut et al., 2012; Caglar Aytac et al., 2015; Aydinuraz et al., 2016; Dayong Hao et al., 2016; Fawzy et al., 2018; Bonte et al., 2019; Li J. et al., 2019). It was 62.92% (6,851/10,888) in the ICSI-AOA group and 38.93% (3,772/9,690) in the non-AOA group. AOA with calcium ionophore significantly raised the fertilization rate of patients re-undergoing ICSI (OR 2.54; 95% CI, 1.52–4.24; *P* = 0.0004; *I*^2^= 98%; Figure 4A). The funnel plot showed obvious publication bias among the included studies (Supplementary Figure 4A). Considering the extremely high heterogeneity, we conducted subgroup analyses. They illustrated that for the “previous fertilization failure or low fertilization rate” subgroup, AOA with calcium ionophore significantly enhanced the fertilization rate (OR 3.77; 95% CI, 2.28–6.23; *P* < 0.00001; *I*^2^= 97%); whereas, for the “embryo developmental problems (embryonic development block or sperm factor or DOR)” subgroup, AOA with calcium ionophore did not influence the fertilization rate (OR 1.18; 95% CI, 0.64–2.14; *P* = 0.60; *I*^2^= 96%; Figure 4B, Supplementary Figure 4B). These results demonstrated a significant association between fertilization rate and types of people included.

#### Cleavage Rate

**Figure 5:**
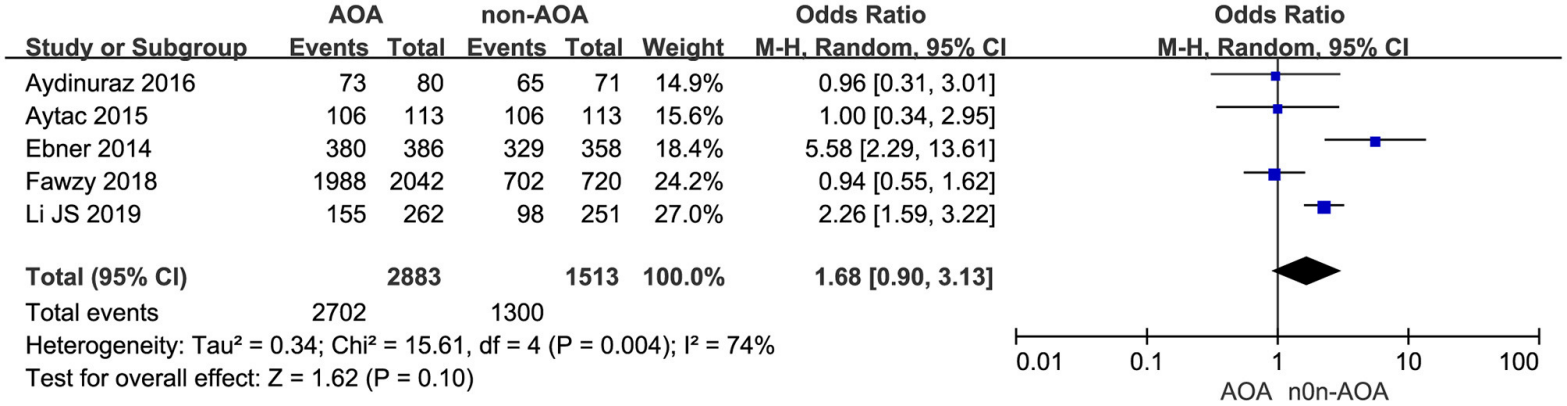
Forest plot of cleavage rate between AOA treatment and non-AOA treatment patient groups using calcium ionophore.

Five studies reported the cleavage rate (Ebner et al., 2014; Caglar Aytac et al., 2015; Aydinuraz et al., 2016; Fawzy et al., 2018; Li J. et al., 2019). It was 93.72% (2,702/2,883) in the AOA treatment group and 85.92% (1,300/1,513) in the control group. Employing the random effects model, this difference was not discovered to be of statistical significance: OR 1.68; 95% CI, 0.90–3.13; *P* = 0.10; *I*^2^= 74% (Figure 5, Supplementary Figure 5). The results indicated that there was no association between cleavage rate and AOA with calcium ionophore treatment.

#### Top-Quality Embryo Rate

**Figure 6:**
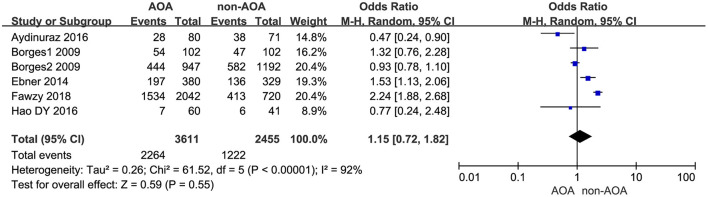
Forest plot of top-quality embryo rate between AOA treatment and non-AOA treatment patient groups using calcium ionophore.

Six studies explored the effect of AOA with calcium ionophore on top-quality embryos (Borges et al., 2009a,b; Ebner et al., 2014; Aydinuraz et al., 2016; Dayong Hao et al., 2016; Fawzy et al., 2018). The top-quality embryo rate was 62.70% (2,264/3,611) in the AOA treatment group and 49.78% (1,222/2,455) in the control group. Using the random effects model, we found AOA with calcium ionophore could not improve the top-quality embryo rate (OR 1.15; 95% CI, 0.72–1.82; *P* = 0.55; *I*^2^= 92%; Figure 6, Supplementary Figure 6).

#### Blastocyst Formation Rate

**Figure 7:**
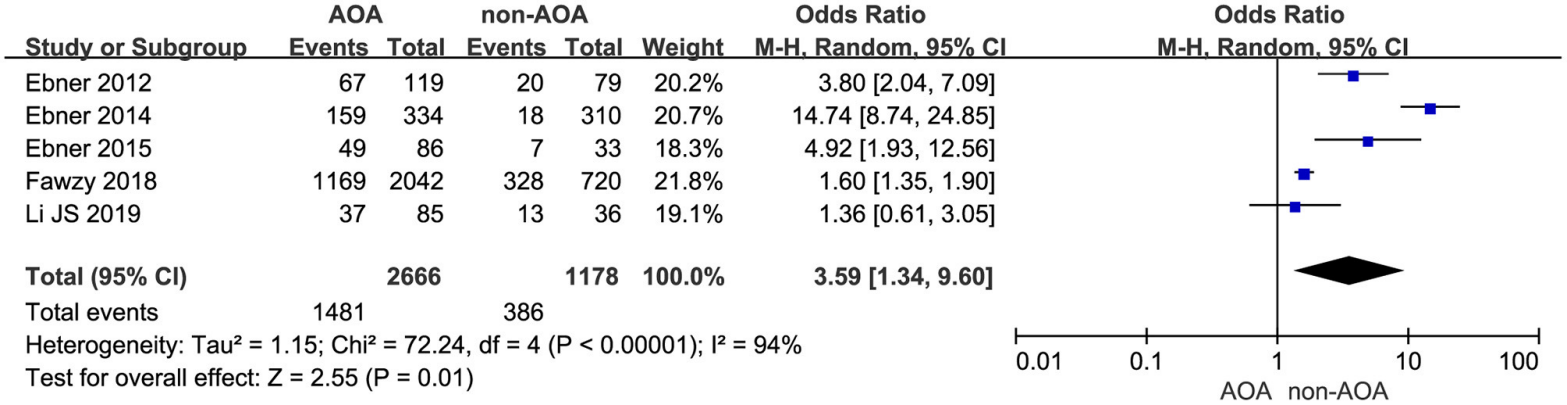
Forest plot of blastocyst formation rate between AOA treatment and non-AOA treatment patient groups using calcium ionophore.

Five studies represented the related information about blastocyst formation (Ebner et al., 2012, 2014, 2015; Fawzy et al., 2018; Li J. et al., 2019). It was 55.55% (1,481/2,666) in the ICSI-AOA group and 32.77% (386/1,178) in the non-AOA group. Applying a random effects model to analyze the blastocyst formation rate, a statistically significant difference was observed: OR 3.59; 95% CI, 1.34–9.60; *P* = 0.01; *I*^2^= 94% (Figure 7, Supplementary Figure 7). The pooled results suggested AOA with calcium ionophore improved the blastocyst formation rate.

#### Implantation Rate

**Figure 8:**
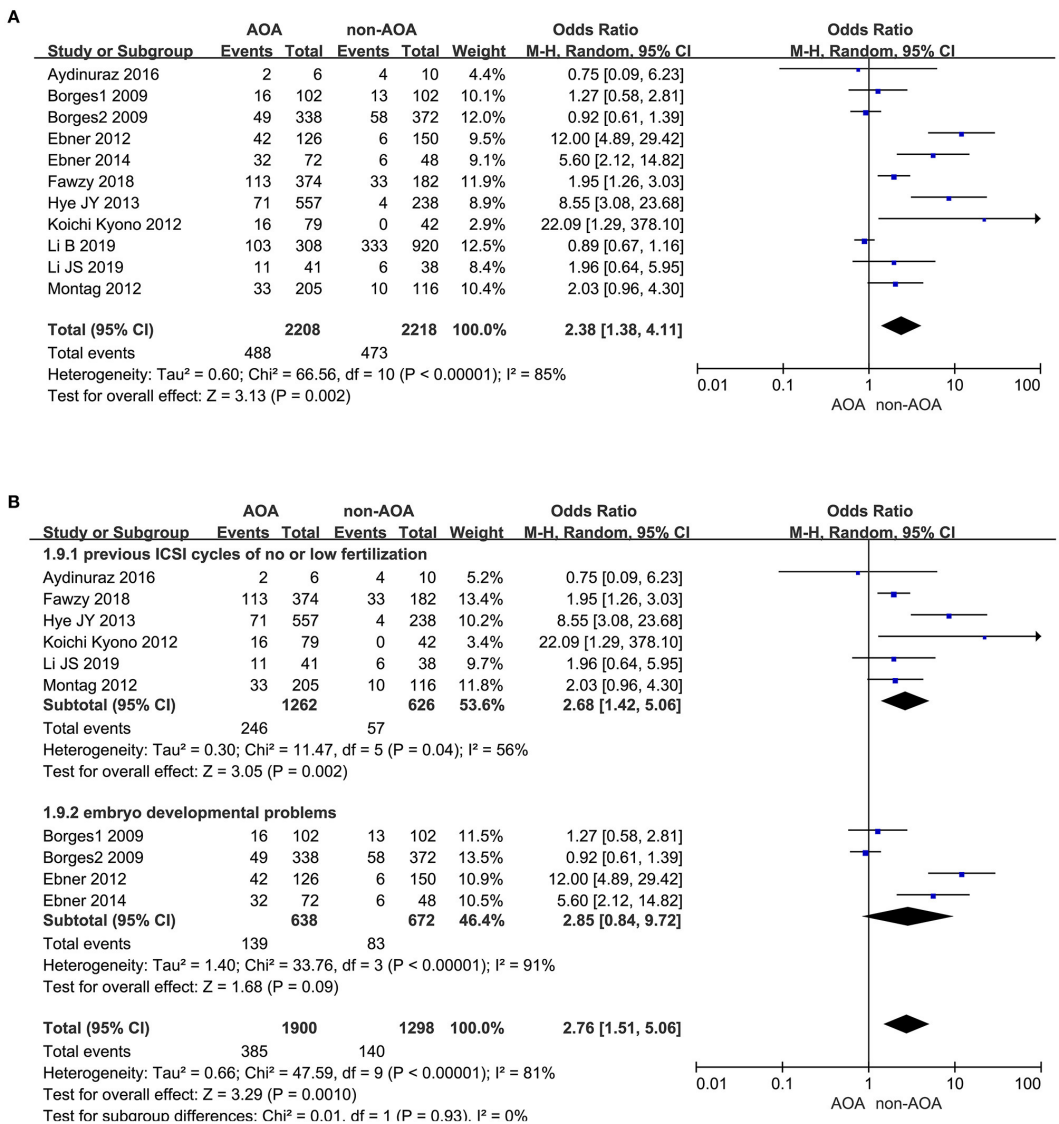
**(A)** Forest plot of implantation rate between AOA treatment and non-AOA treatment patient groups using calcium ionophore. **(B)** The subgroup analysis of implantation rate between AOA treatment and non-AOA treatment patient groups.

Eleven articles presented the implantation rate, of which there were 488 (488/2,208) implanted embryos in the AOA with calcium ionophore group and 473 (473/2,218) implanted embryos in the non-AOA group (Borges et al., 2009a,b; Ebner et al., 2012, 2014; Kyono et al., 2012; Montag et al., 2012; Yoon et al., 2013; Aydinuraz et al., 2016; Fawzy et al., 2018; Li B. et al., 2019; Li J. et al., 2019). A statistically significant difference of implantation rate was observed between ICSI-AOA and non-AOA groups. In view of the high heterogeneity (*P* < 0.00001; *I*^2^= 85%), we derived the pooled OR with a random effects model (OR 2.38; 95% CI, 1.38–4.11; *P* = 0.002; *I*^2^= 85%; Figure 8A). The funnel plot remained skewed which suggested publication bias (Supplementary Figure 8A). Given the remarkable heterogeneity, subgroup analyses were conducted. For the “previous fertilization failure or low fertilization rate” subgroup, AOA with calcium ionophore obviously increased its implantation rate (OR 2.68; 95% CI, 1.42–5.06; *P* = 0.002; *I*^2^= 56%); however, for the “embryo developmental problems (embryonic development block or sperm factor or DOR)” subgroup, AOA with calcium ionophore could not improve the implantation rate (OR 2.85; 95% CI, 0.84–9.72; *P* = 0.09; *I*^2^= 91%; Figure 8B, Supplementary Figure 8B). For the “previous fertilization failure or low fertilization rate” subgroup, AOA in combination with ICSI could improve the implantation rate significantly, but for the “embryo developmental problems (embryonic development block or sperm factor or DOR)” subgroup, AOA could not increase the implantation rate. These results suggested that the high heterogeneity between studies might be due to the different populations included in the study.

#### Miscarriage Rate

**Figure 9:**
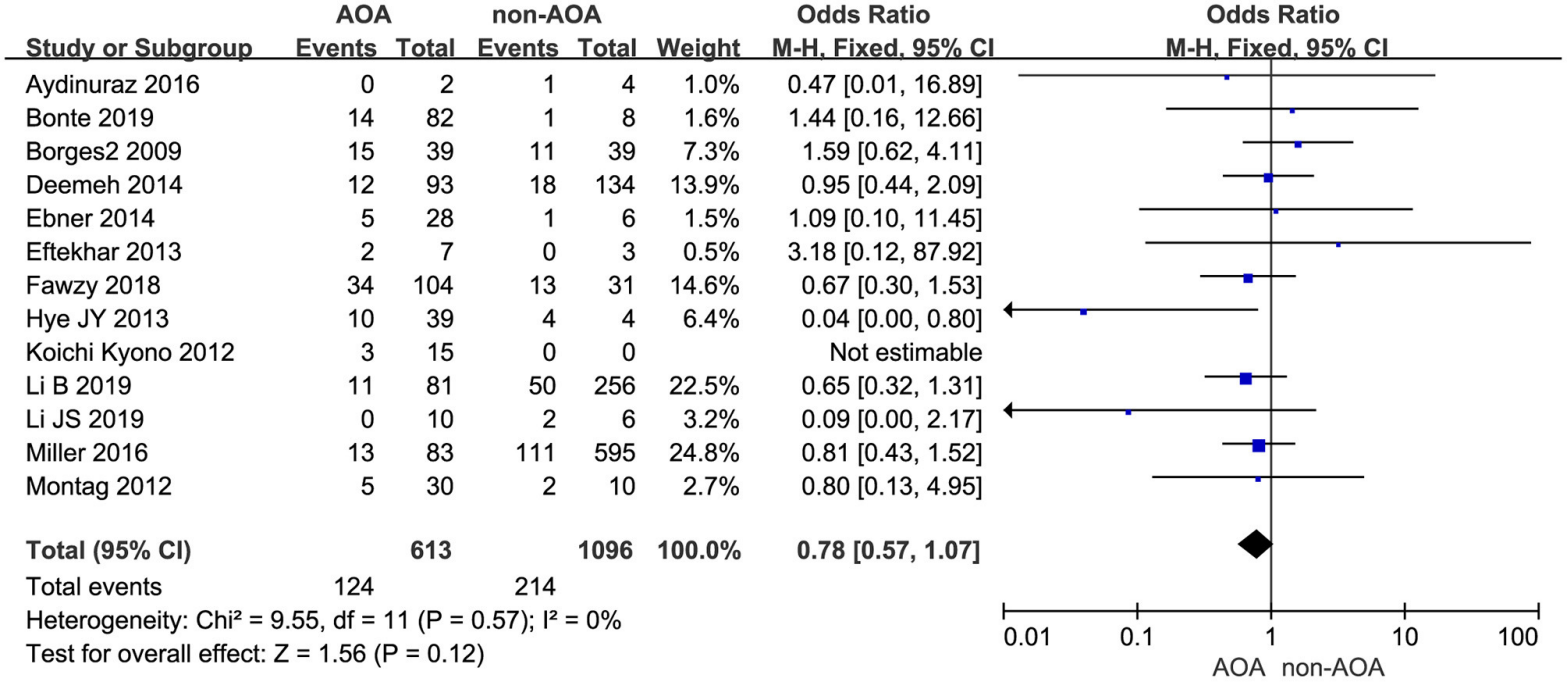
Forest plot of miscarriage rate between AOA treatment and non-AOA treatment patient groups using calcium ionophore.

Thirteen of the included studies investigated the effect of AOA with calcium ionophore on pregnancy outcome (Borges et al., 2009b; Kyono et al., 2012; Montag et al., 2012; Eftekhar et al., 2013; Yoon et al., 2013; Deemeh et al., 2014; Ebner et al., 2014; Aydinuraz et al., 2016; Miller et al., 2016; Fawzy et al., 2018; Bonte et al., 2019; Li B. et al., 2019; Li J. et al., 2019). Among the included infertility patients, 20.23% (124 of 613) of patients in the ICSI-AOA group and 19.53% (214 of 1,096) of patients in control group experienced miscarriage. Using a fixed effects model, we did not discover a statistically significant difference between these two groups (OR 0.78; 95% CI, 0.57–1.07; *P* = 0.12; *I*^2^= 0; Figure 9). Hence, AOA with calcium ionophore did not increase the miscarriage rate. Based on the Begg's test, the *P*-value equaled 0.945 > 0.05, which implied no indication of asymmetry among the included 13 studies (Supplementary Figure 9).

#### Congenital Birth Defects

**Figure 10:**
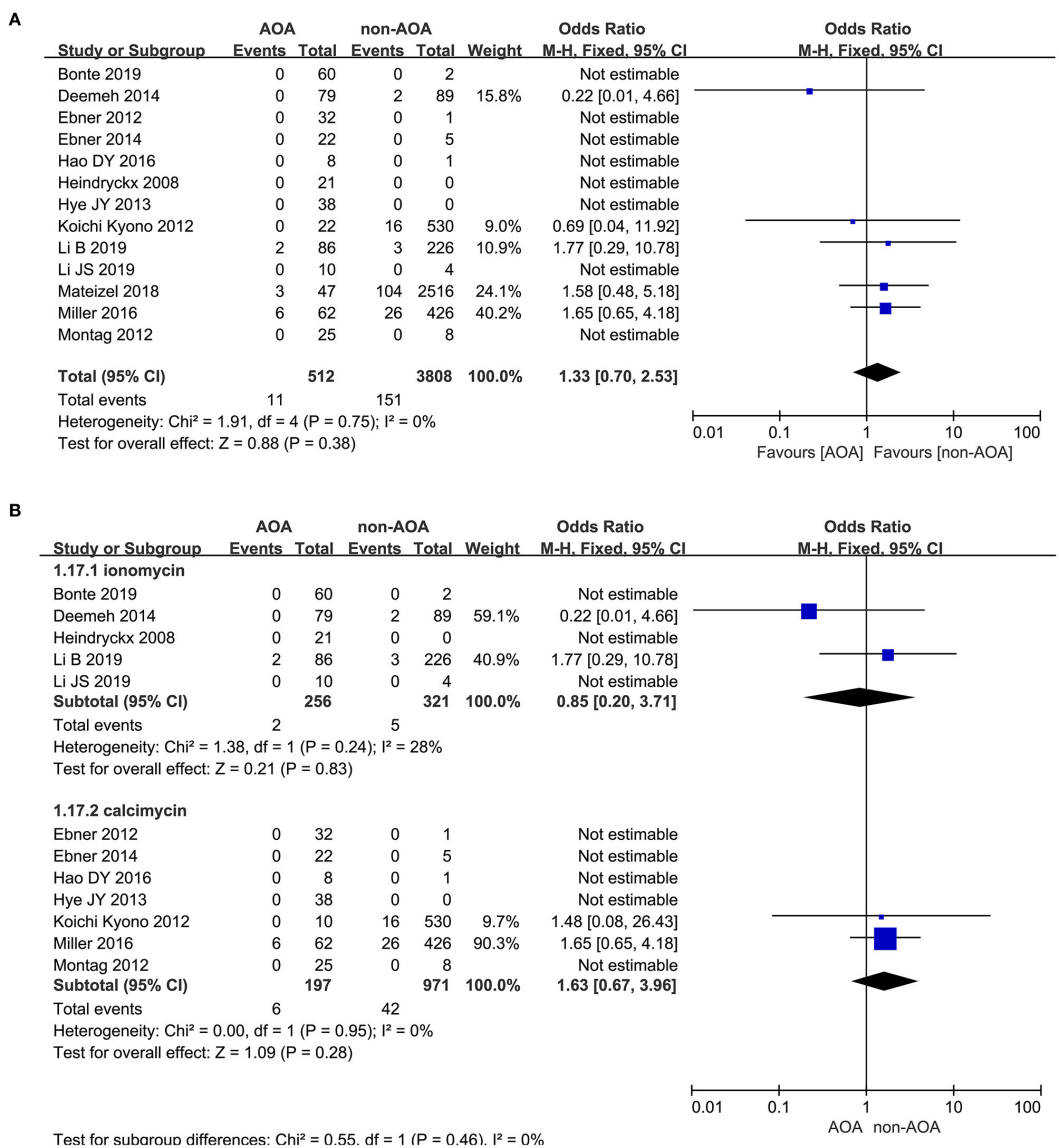
**(A)** Forest plot of congenital birth defect rate between AOA treatment and non-AOA treatment patient groups using calcium ionophore. **(B)** The subgroup analysis of birth defect rate between AOA treatment and non-AOA treatment patient groups.

Thirteen out of 22 studies reported the rate of congenital birth defects (Heindryckx et al., 2008; Ebner et al., 2012, 2014; Kyono et al., 2012; Montag et al., 2012; Yoon et al., 2013; Deemeh et al., 2014; Dayong Hao et al., 2016; Miller et al., 2016; Mateizel et al., 2018; Bonte et al., 2019; Li B. et al., 2019; Li J. et al., 2019). The incidence of birth defects was 2.15% (11/512) in the ICSI-AOA treatment group and 3.97% (151/3,808) in the non-AOA control group. According to this meta-analysis, no significant difference was found between the ICSI-AOA group and only ICSI group in congenital anomalies (OR 1.33; 95% CI, 0.70–2.53; *P* = 0.38; *I*^2^= 0; Figure 10A). The result suggested that AOA with calcium ionophore did not affect the safety of offspring. Additionally, funnel plots (symmetric) and Egger's test illustrated no obvious publication bias (Supplementary Figure 10A). Furthermore, subgroup comparison of different activators between ionomycin and calcimycin were conducted. In the “ionomycin” subgroup (Heindryckx et al., 2008; Deemeh et al., 2014; Bonte et al., 2019; Li B. et al., 2019; Li J. et al., 2019), congenital birth defects occurred in 0.78% (2/256) of patients in the ICSI-AOA group and 1.6% (5/312) in the non-AOA group, respectively. Also in this meta-analysis, no significant difference was found in the ionomycin subgroup (OR 0.85; 95% CI, 0.20–3.71; *P* = 0.85; *I*^2^= 28%; Figure 10B). In the “calcimycin” subgroup (Ebner et al., 2012, 2015; Kyono et al., 2012; Montag et al., 2012; Yoon et al., 2013; Dayong Hao et al., 2016; Miller et al., 2016), no significant difference was found between the ICSI-AOA group (3.05%, 6/197) and non-AOA group (4.33%, 42/971) (OR 1.63; 95% CI, 0.67-3.96; *P* = 0.28; *I*^2^= 0; Figure 10B). These results suggested that using different types of calcium ionophore (ionomycin and calcimycin) for AOA would not increase the incidence of birth defects in the offspring of infertile patients. The study of Mateizel et al. (2018) was excluded due to four newborns resulting from mixed transplantation of calcimycin and ionomycin activation cycles. Another research study was excluded, in which only 10 newborns after calcimycin treatment were included, and no birth defects occurred (Kyono et al., 2012). After SrCl_2_ activation treatment, 12 babies were born without birth defects, so they were not included in the subgroup analysis. The funnel plot is shown in Supplementary Figure 10B.

#### Neonatal Sex Ratio

**Figure 11:**
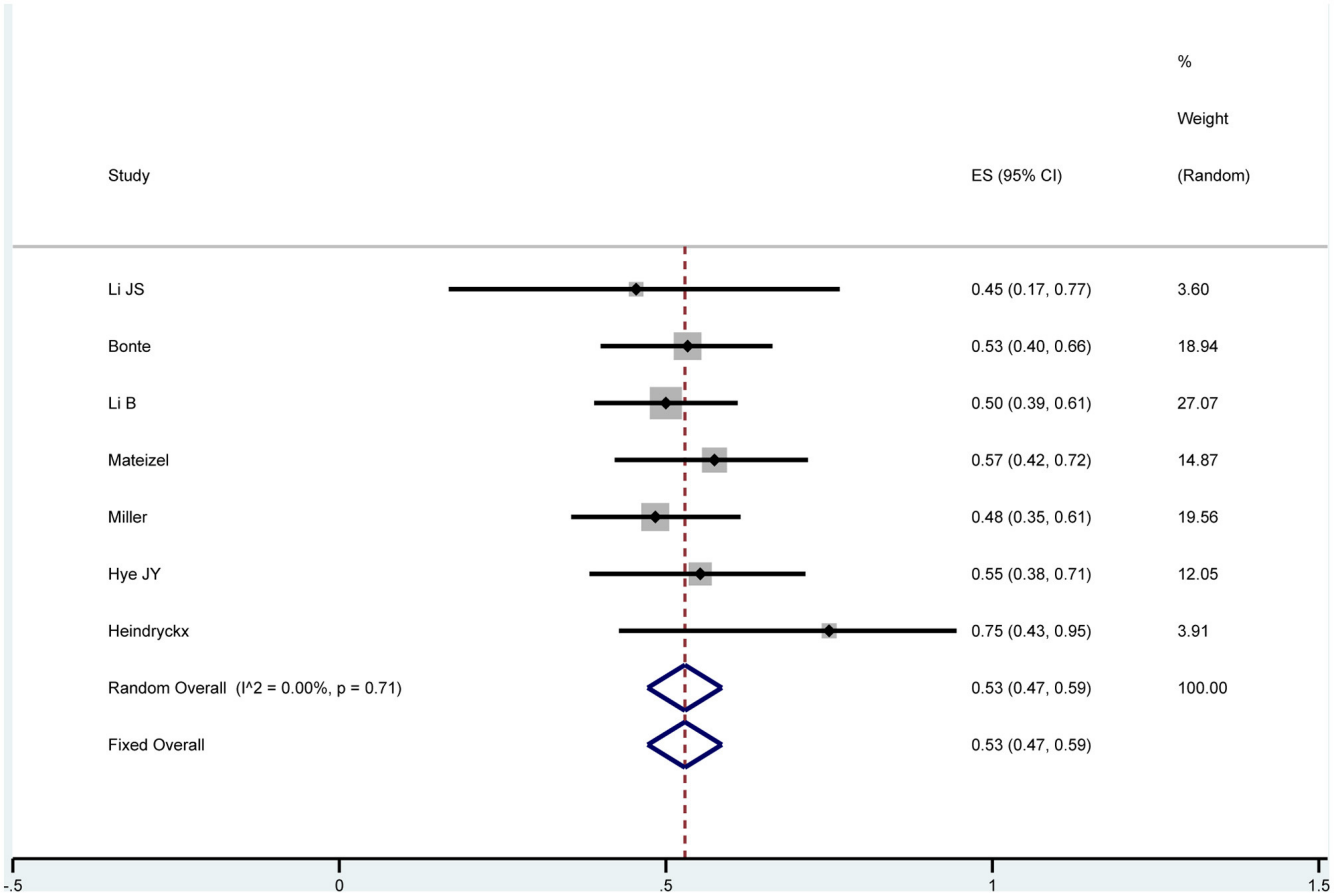
Forest plots of the included studies reporting data on sex ratio.

Seven studies reported the neonatal sex and were analyzed by one-arm analysis (Heindryckx et al., 2008; Yoon et al., 2013; Miller et al., 2016; Mateizel et al., 2018; Bonte et al., 2019; Li B. et al., 2019; Li J. et al., 2019). After undergoing AOA with calcium ionophore treatment, 167 boys and 149 girls were born. Using a fixed effects model, no significant difference was discovered: OR 0.53; 95% CI, 0.47–0.59; *P* = 0.71; *I*^2^= 0 (Figure 11). There was no evidence of publication bias in the neonatal sex ratio (Supplementary Figure 11). These above results revealed that AOA with calcium ionophore had no effect on the sex ratio of newborns.

## Discussion

### Summary of Findings

Calcium ionophore treatment during AOA is a common chemical activation method and is always used in cases with low fertilization rates or developmental block. Several studies showed that the use of AOA with calcium ionophore treatment improved pregnancy and live birth rate to a certain degree. As the number of studies increase, it is necessary to find more reliable evidence of calcium ionophore in improving pregnancy outcome. In this study, a meta-analysis was performed to explore the efficacy and safety of calcium ionophore treatment during AOA in improving pregnancy outcome and decreasing the incidence of congenital birth defects.

This meta-analysis was conducted based on five RCTs and 17 observational studies. Two publications (Kyono et al., 2012; Fawzy et al., 2018) dealt with SrCl_2_, which is not a regular Ca-ionophore but is thought to use calcium channels since Ca^2+^ and Sr^2+^ are very similar molecules. Recently, Lu et al. (2018) found that SrCl_2_ activation cannot stimulate calcium oscillation or parthenogenic division in matured oocytes *in vitro* or those with smooth endoplasmic reticulum. Therefore, these two publications referring to SrCl_2_ were also included. One study (Ebner et al., 2014) reported that Ca^2+^-ionophore was meant to activate mitosis at the cleavage stage and not activate the oocyte, which was controversial but we still included the study. The only fact is that both classical AOA and stimulation of mitosis have the same underlying causative effect which is a reduced/depleted intracellular calcium level. Luckily, Ebner et al. did apply the Ca^2+^-ionophore stimulus exactly at the same time for both indications (classical AOA and stimulation of mitosis) according to their ethical vote, so that the application mode was exactly as all other studies mentioned. The pregnancy rate in 17 studies showed it was also higher in the ICSI-AOA group (34.87%, 649/1,861) than in the non-AOA group (33.68%, 799/2,372). Only 14 studies reported the effect of AOA with calcium ionophore on live birth rate, and it was higher in the ICSI-AOA group (28.90%, 446/1,543) than in the non-AOA group (26.13%, 543/2,078). Focusing on the primary outcomes, calcium ionophore in combination with ICSI significantly increased the pregnancy rate and live birth rate. The secondary outcomes reported that AOA with calcium ionophore played a positive role in improving fertilization, blastulation, and implantation rate, which suggested that AOA with calcium ionophore was effective for oocyte activation. The results of subgroup analysis of the fertilization rate suggested that for patients in the “previous fertilization failure or low fertilization rate” subgroup, in general, AOA significantly increased the fertilization rate of patients, but could not improve the fertilization rate of patients with embryo developmental problems. Similarly, the implantation rate had the same results as the fertilization rate. And AOA did not enhance the cleavage and top-quality embryo rate. Moreover, calcium ionophore in combination with ICSI did not improve the incidence of miscarriage, congenital birth defects, and neonatal sex ratio, which suggested that AOA with calcium ionophore is safe for oocyte activation.

### Biological and Clinical Significance

Conventional IVF resulted in low fertilization rates or even no fertilization. The most likely cause is oocyte activation failure, followed by few or poor quality of sperm or oocyte characteristics. ICSI is widely used to treat infertility caused by male factors, but low fertilization and complete fertilization failure still arise in a few special infertility populations due to abnormal cell mitosis and embryo cleavage. Calcium has a major role in cell mitosis and embryo cleavage (Swanson et al., 1997; Goud et al., 1999; Tosti, 2006). Inadequate physiological or artificial calcium causes cleavage anomalies or embryo arrest. Multiple methods were proved to solve activation/fertilization failure problems after ICSI, among which calcium ionophore is advantageous and has no evidence of toxicity or harmful outcome (Miller et al., 2016). In addition, ICSI in combination with AOA by calcium ionophore has mitigated the above infertility problem. Multiple studies reported that oocyte fertilization that failed after ICSI was subsequently activated by calcium ionophore, and the activated oocytes developed normally (Kashir et al., 2010). However, the safety and efficacy of AOA with calcium ionophore are still a routine trepidation. Hence, large-sample clinical studies and meta-analyses or reviews need to be investigated. Recently, two related meta-analyses that primarily concentrated on the efficacy of AOA were reported (Sfontouris et al., 2015; Murugesu et al., 2017). The results demonstrated that fertilization, blastulation, implantation, and clinical pregnancy and live birth rate increased with statistical significance in infertility patients treated with calcium ionophore. Calcium ionophore appears to be the most popular method in oocyte activation. With the increase of AOA-related articles, a new meta-analysis was needed to discuss the safety and efficacy of AOA with calcium ionophore. In this study, we included 22 studies and mainly explored the efficiency and safety of AOA with calcium ionophore in the assisted reproductive field. Our results indicated that AOA with calcium ionophore not only significantly increased the fertilization, blastulation, implantation, and clinical pregnancy and live birth rate, but also did not affect the incidence of miscarriage, congenital birth defects, and neonatal sex ratio.

Inconsistent types of included studies may limit the possibility in obtaining a more reliable conclusion and cause high heterogeneity. There were five RCTs, nine prospective observational trials, and eight retrospective trials in this study. More RCT studies promoted a creditable outcome. Fertilization failure after ICSI was uncommon, leading to difficultly in designing and conducting a methodologically precise RCT, so a small number of RCT studies were included in this study. Our results tended to support the notion that calcium ionophore treatment was safe and effective. The analysis results indicated that AOA with calcium ionophore resulted in a statistically significant improvement in live birth rate. The degree of heterogeneity was high and publication bias appeared. Subsequently, we performed a sensitivity analysis to discover the cause of heterogeneity in the 14 studies. The results indicated that five low-quality studies (Ebner et al., 2012; Deemeh et al., 2014; Kang et al., 2015; Bonte et al., 2019; Li B. et al., 2019) had a great influence on heterogeneity. After excluding these five low-quality studies, the heterogeneity and publication bias disappeared among those remaining nine studies. The meta analysis of the remaining nine studies also suggested AOA with calcium ionophore significantly increased the live birth rate. Through comprehensively analyzing the causes of bias, we found that two studies (Ebner et al., 2012; Bonte et al., 2019) covered pre- and post-control of their own case involving patients with fertilization failure or low fertilization rate in the previous cycle, and the live birth rate with the treatment of calcium ionophore in the next cycle was significantly higher than those without calcium ionophore in the previous cycle. Another two studies (Deemeh et al., 2014; Li B. et al., 2019) showed that AOA with calcium ionophore could improve the birth rate of patients with previous fertilization failure or low fertilization rate, as well as patients with testicular sperm extraction (TESE) or severe teratozoospermia. However, Kang et al. (2015) thought that patients with previous fertilization failure or low fertilization rate could not achieve the same birth rate as those who received TESE when they were treated with TESE again. Therefore, there is heterogeneity in the inclusion and evaluation for live birth rate in these five studies.

Furthermore, in subgroup analyses, consistent results where AOA with calcium ionophore significantly increased the birth rate of re-assisted pregnancy were found in the “previous fertilization failure or low fertilization rate” subgroup (OR 4.76; 95% CI, 2.01–11.25; *P* = 0.0004; *I*^2^= 65%) and “embryo developmental problems (embryonic development block or sperm factor or DOR)” subgroup (OR 4.59; 95% CI, 1.35–15.65; *P* = 0.01; *I*^2^= 72%). Hence, the high heterogeneity in this part was caused by the design and quality of studies but not the fact that the included studies recruited diverse study populations. High clinical heterogeneity could be explained by patient characteristics, fertility treatment plans of different centers, and distinct inclusion and exclusion criteria. After eliminating specific studies by sensitivity analysis, our findings were collated by a mixture of random and fixed models. Moreover, the statistical heterogeneity could be handled by subgroup analysis. And subgroup analysis did not change our results. It increased the credibility of the findings in AOA with calcium ionophore promoting live birth rate. The overall pregnancy rate was significantly increased in the ICSI-AOA group. And no publication bias appeared. After eliminating specific studies by sensitivity analysis, we found high heterogeneity appearing among these 17 included studies. Aiming to analyze the reasons for high heterogeneity, subgroup analysis was performed. The results demonstrated that AOA with calcium ionophore uncommonly enhanced the pregnancy rate in both subgroups. It ensured more credible results in AOA with calcium ionophore promoting overall pregnancy rate.

For the secondary results, in view of the fact that we were more interested in the safety of AOA for offspring, the analysis results indicated that AOA with calcium ionophore not only reduced the incidence of congenital birth defects, but also a decrease was seen in the ICSI-AOA group. Furthermore, the degree of heterogeneity was low. However, although 13 studies were included, 8 studies had few newborns (Heindryckx et al., 2008; Ebner et al., 2012, 2015; Montag et al., 2012; Yoon et al., 2013; Dayong Hao et al., 2016; Bonte et al., 2019; Li J. et al., 2019). Therefore, increasing the number of newborns contributed to a more reliable conclusion in assessing the safety of AOA with calcium ionophore. Similar results were found in miscarriage rate. AOA with calcium ionophore did not affect the pregnancy outcome and a similar miscarriage rate was discovered in the ICSI-AOA group and non-AOA group. Thirteen articles mentioned miscarriage rate, among which one study reported that no clinical pregnancy occurred in the only-ICSI group. The number of research articles and cases was acceptable, and low heterogeneity and no publication bias occurred. Therefore, the conclusion that AOA with calcium ionophore did not promote miscarriage rate was evidence-based. Moreover, the fertilization, blastocyst formation, and implantation rate all improved with a statistical difference. However, in the subgroup analyses, ICSI-AOA could obviously improve the fertilization and implantation rate of patients with “previous fertilization failure or low fertilization rate,” but not the patients with “embryo developmental problems.” It reminded us that it was of little significance to use calcium ionophore during AOA to activate oocytes for patients with “embryo developmental problems.” Compared with the non-AOA group, no evidence in improving cleavage and top-quality embryo rate was found, but a trend of promotion appeared. The gender of the fetus at birth was also of concern to us. The results revealed that AOA with calcium ionophore had no effect on the sex ratio of newborns using by one-arm analysis. This study did not pay attention to the baby's development in the future, and ignored whether gene expression was affected by treatment by calcium ionophore.

## Conclusion

These existing studies proved that AOA by calcium ionophore following ICSI not only significantly improved live birth, overall pregnancy, implantation, fertilization, and blastocyst formation rates, but also did not affect the incidence of miscarriage and congenital birth defects. Strong evidence for the efficacy and safety of calcium ionophore application in improving oocyte activation has been discovered. This meta-analysis indicated that using calcium ionophore to activate oocytes was beneficial for couples with poor fertilization rates following ICSI. We would suggest using AOA with calcium ionophore in routine practice for failed fertilization following ICSI.

## Data Availability Statement

The original contributions presented in the study are included in the article/Supplementary Material, further inquiries can be directed to the corresponding author/s.

## Author Contributions

YS designed the paper. HZ wrote the manuscript. YS and DZ drew the figures. JW, DZ, and YC revised and supervised the project. HB contributed to supervision and reviewed the manuscript. All authors read and approved the final manuscript.

## Funding

This work was supported by the Technology Plan of Yantai (Grant no. 2020MSGY089), the National Natural Science Foundation of China (Grant no. 82101698), and the Science and the Natural Science Foundation of Shandong Province (Grant no. ZR2017PH047).

## Conflict of Interest

The authors declare that the research was conducted in the absence of any commercial or financial relationships that could be construed as a potential conflict of interest.

## Publisher's Note

All claims expressed in this article are solely those of the authors and do not necessarily represent those of their affiliated organizations, or those of the publisher, the editors and the reviewers. Any product that may be evaluated in this article, or claim that may be made by its manufacturer, is not guaranteed or endorsed by the publisher.

## Supplementary Material

Supplementary Table 1 NOS for the risk of bias and the quality assessment of included studies.

Supplementary Figure 1 Risk of bias of the included RCTs.

Supplementary Figure 2 **(A)** Funnel plots of publication bias in pregnancy rate. **(B)** Funnel plots of publication bias in pregnancy rate subgroup analysis.

Supplementary Figure 3 **(A)** Funnel plots of publication bias in live birth rate. **(B)** Funnel plots of publication bias in live birth rate subgroup analysis.

Supplementary Figure 4 **(A)** Funnel plots of publication bias in fertilization rate. **(B)** Funnel plots of publication bias in fertilization rate subgroup analysis.

Supplementary Figure 5 Funnel plots of publication bias in cleavage rate.

Supplementary Figure 6 Funnel plots of publication bias in top-quality blastocyst rate.

Supplementary Figure 7 Funnel plots of publication bias in blastocyst formation rate.

Supplementary Figure 8 **(A)** Funnel plots of publication bias in implantation rate. **(B)** Funnel plots of publication bias in implantation rate subgroup analysis.

Supplementary Figure 9 Funnel plots of publication bias in miscarriage rate.

Supplementary Figure 10 **(A)** Funnel plots of publication bias in congenital birth defect rate. **(B)** Funnel plots of publication bias in congenital birth defect rate subgroup analysis.

Supplementary Figure 11 Funnel plots of publication bias in sex ratio.
