# Supplementary material for: Assisted Oocyte Activation With Calcium Ionophore Improves Pregnancy Outcomes and Offspring Safety in Infertile Patients: A Systematic Review and Meta-Analysis
Source: Front Physiol. 2022 Jan 24;12:751905. doi: 10.3389/fphys.2021.751905 (PMC8819094; doi:10.3389/fphys.2021.751905)
Supplement: Supplementary Table 1 — NOS for the risk of bias and the quality assessment of included studies. [file Table_1.pdf]

Supplemental table 1. NOS for the risk of bias and the quality assessment of included studies

Author	Year	Selection	Comparability	Outcome	Total score
Li JS	2019	★★★★	★★	★★★	9
Bonte	2019	★★★	★	★★★	7
Li B	2019	★★	★★	★★★	7
Mateizel	2018	★★		★★★	5
Miller	2016	★★	★★	★★★	7
Aydinuraz	2016	★★★★	★★	★★★	9
Hao DY	2016	★★★★	★★	★★★	9
Ebner	2015	★★★★	★	★★★	8
Hee JK	2015	★★	★	★★★	6
Ebner	2014	★★★★	★★	★★★	9
Mateizel	2014	★★	★	★★★	6
Hye JY	2013	★★★	★	★★★	7
Ebner	2012	★★★★	★★	★★★	9
Montag	2012	★★★	★★	★★★	8
Meerschaut,	2012	★★★★	★★	★★★	9
Koichi Kyono,	2012	★★★	★	★★★	7
Heindryckx,	2008	★★★★	★	★★★	8
