# Supplementary material for: Assisted Oocyte Activation With Calcium Ionophore Improves Pregnancy Outcomes and Offspring Safety in Infertile Patients: A Systematic Review and Meta-Analysis
Source: Front Physiol. 2022 Jan 24;12:751905. doi: 10.3389/fphys.2021.751905 (PMC8819094; doi:10.3389/fphys.2021.751905)

Supplemental Fig. 1

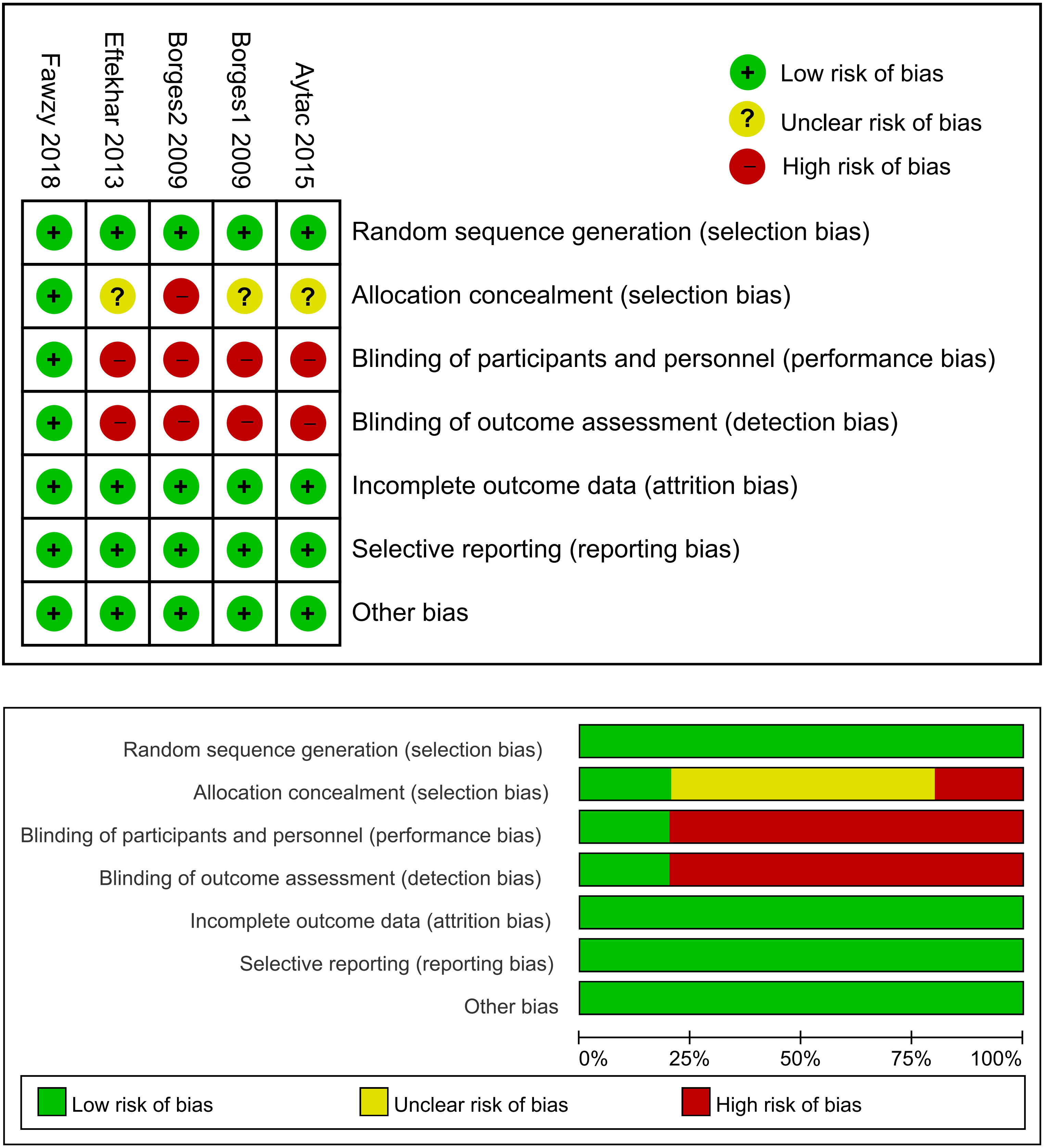

Supplemental Fig. 1: Risk of bias of the included RCTs.

Supplemental Fig. 2A

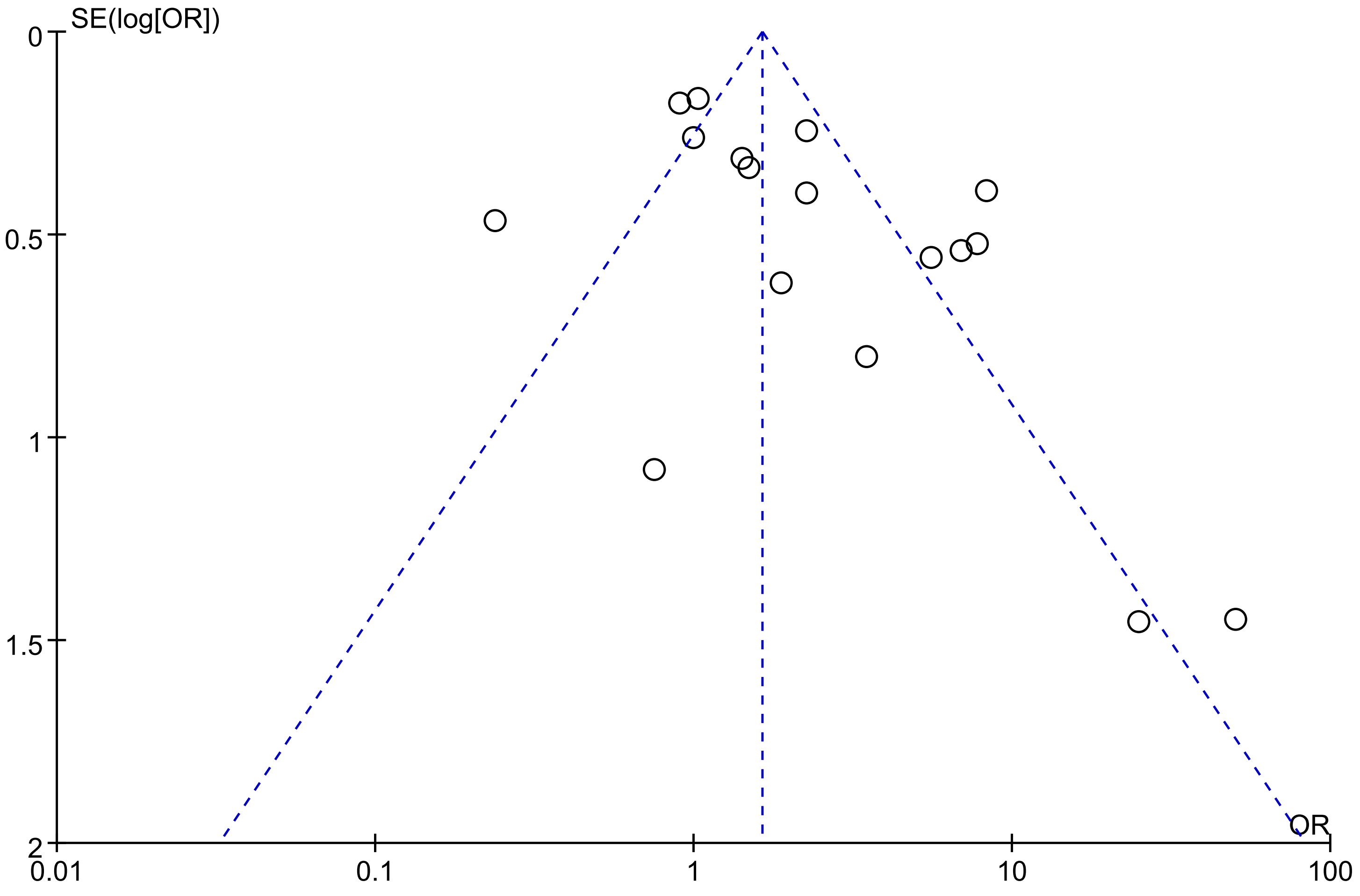

Supplemental Fig. 2B

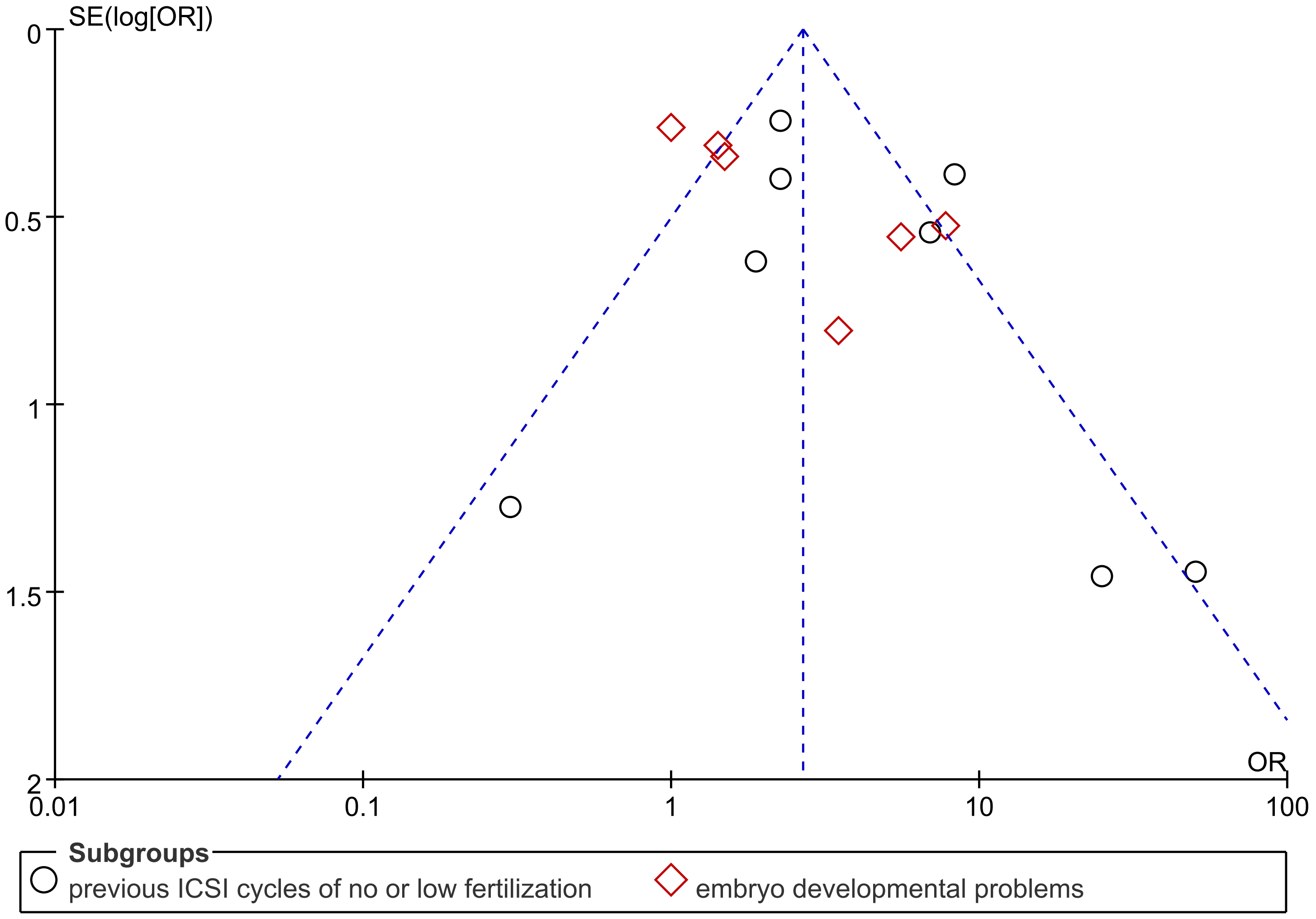

Supplemental Fig. 3A

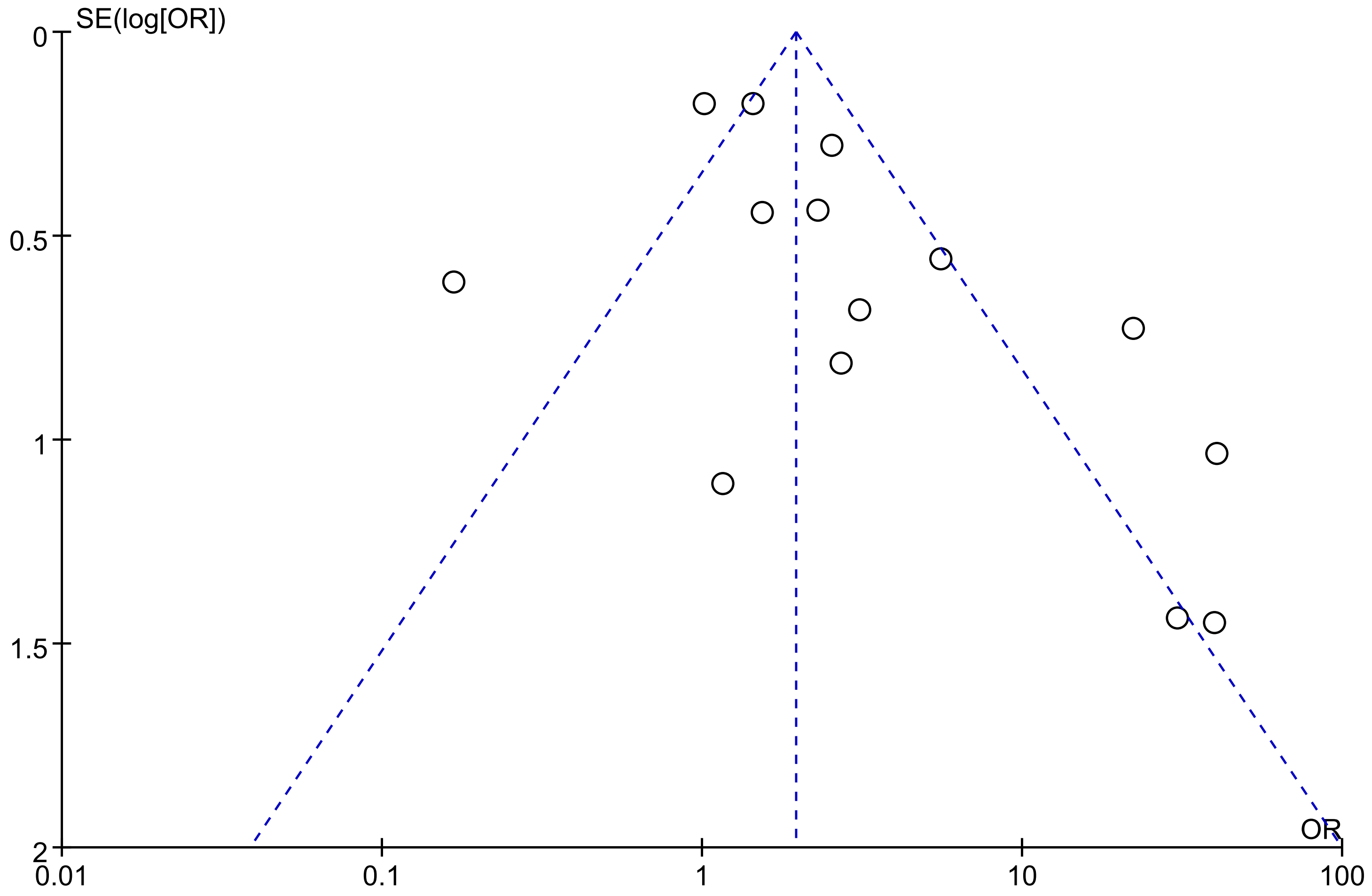

Supplemental Fig. 3B

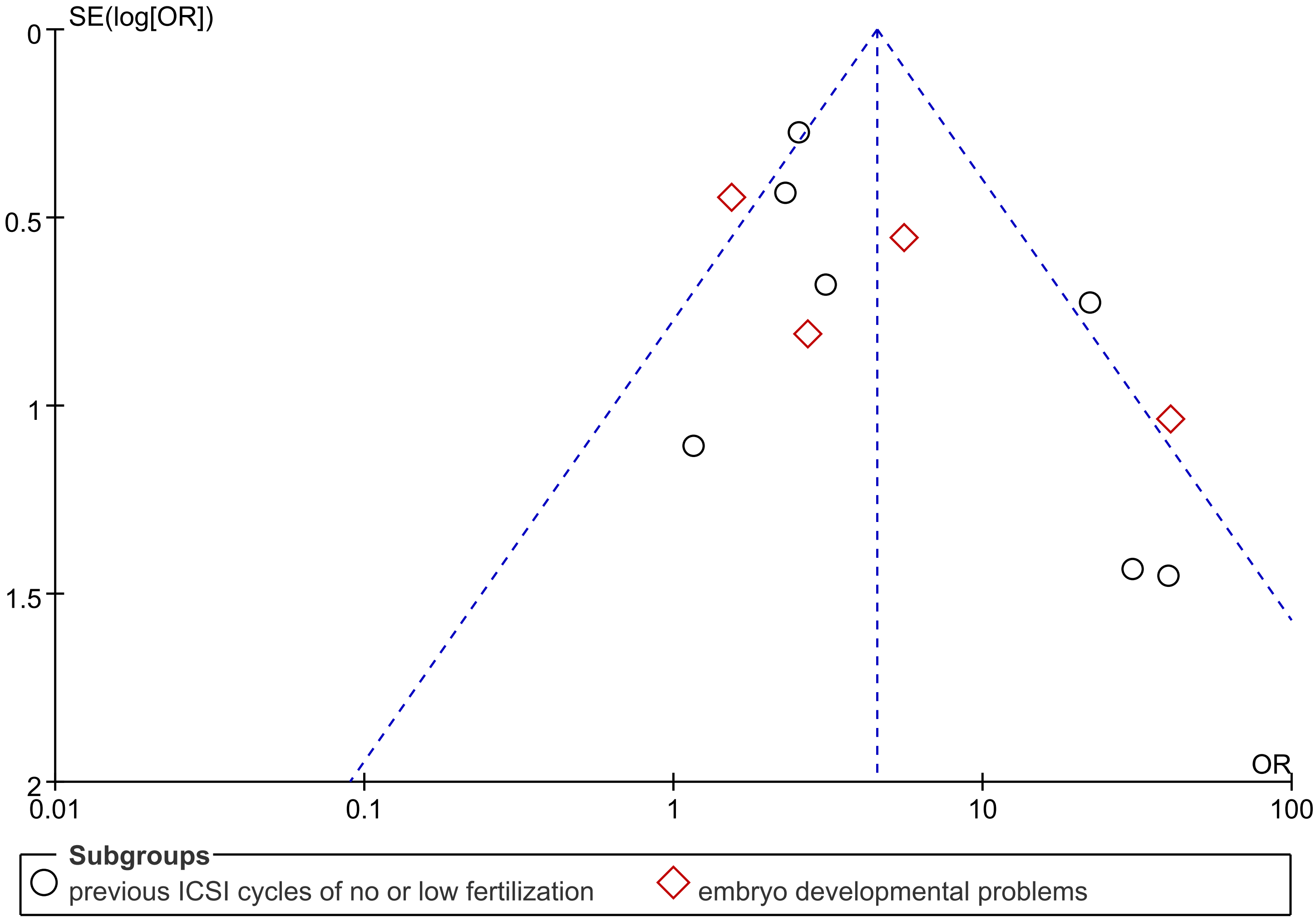

Supplemental Fig. 4A

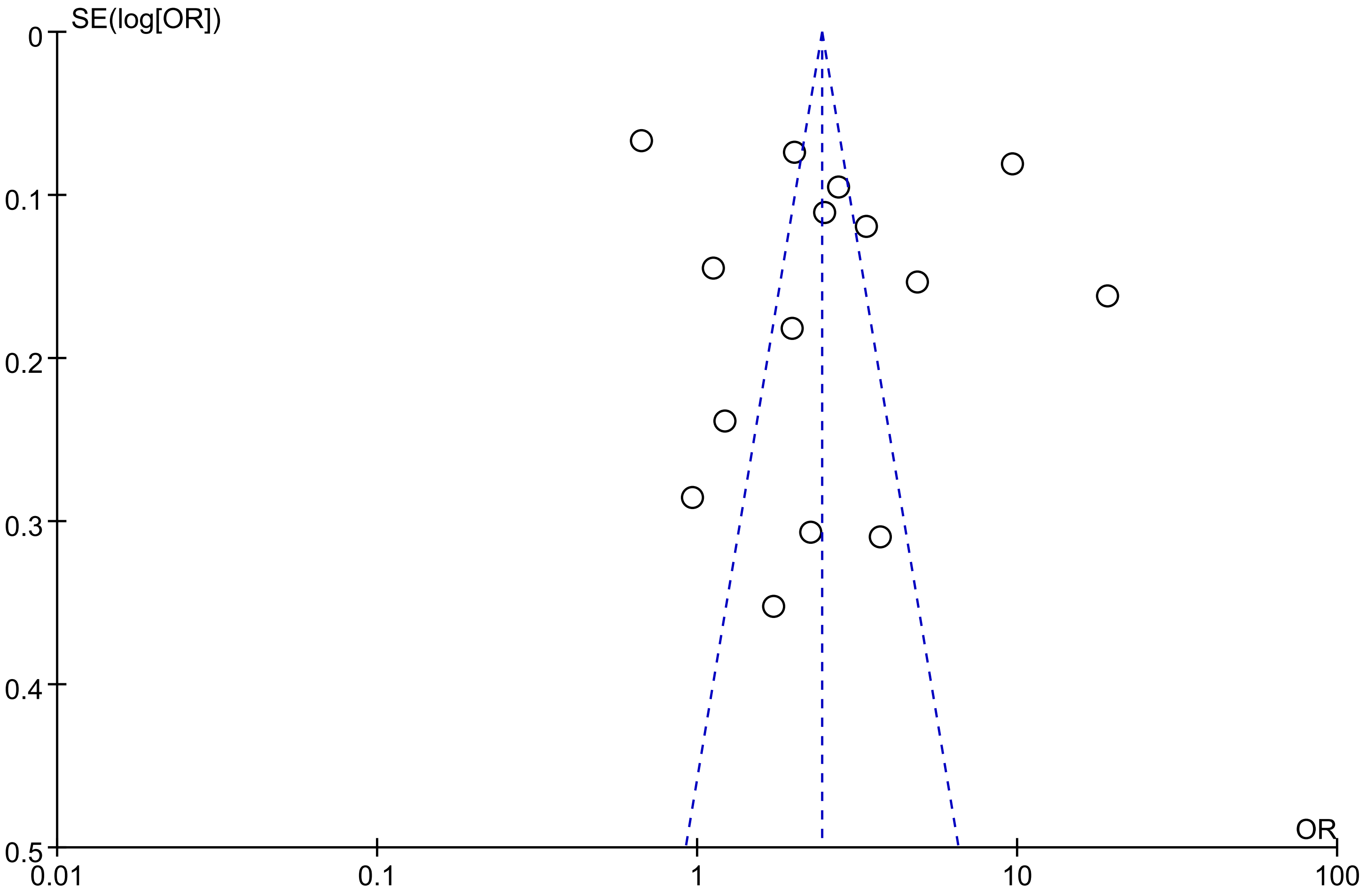

Supplemental Fig. 4B

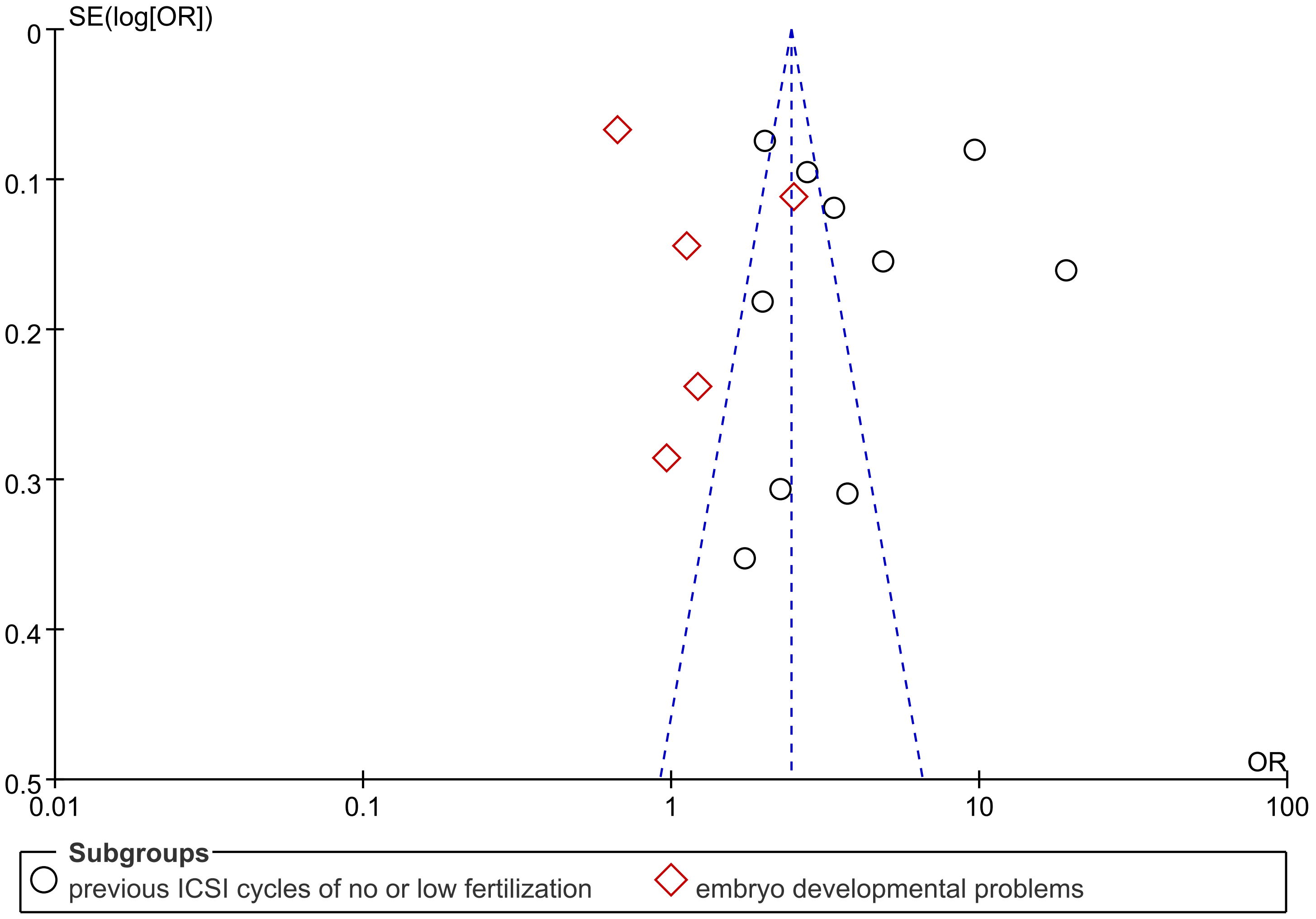

Supplemental Fig. 5

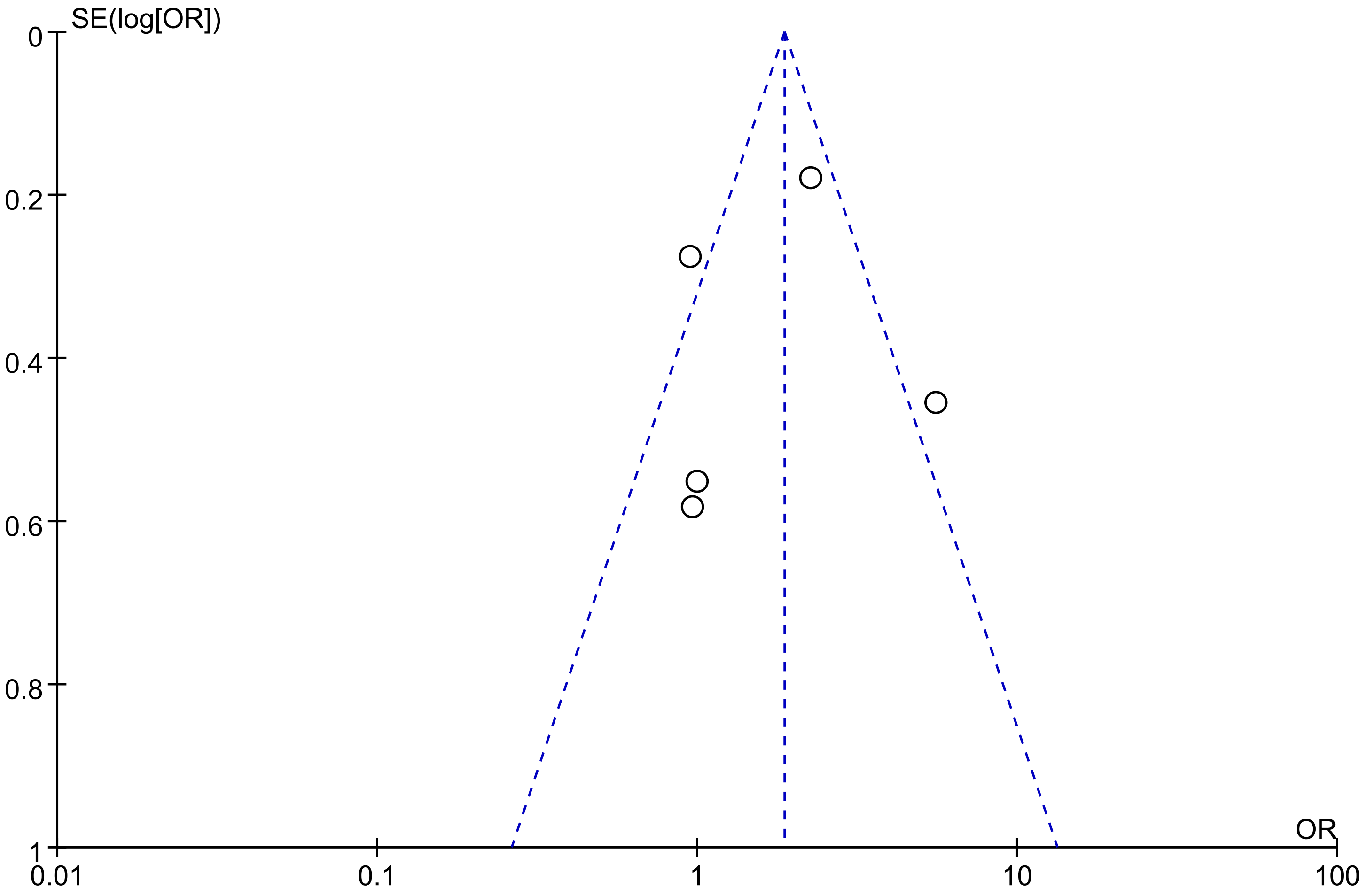

Supplemental Fig. 6

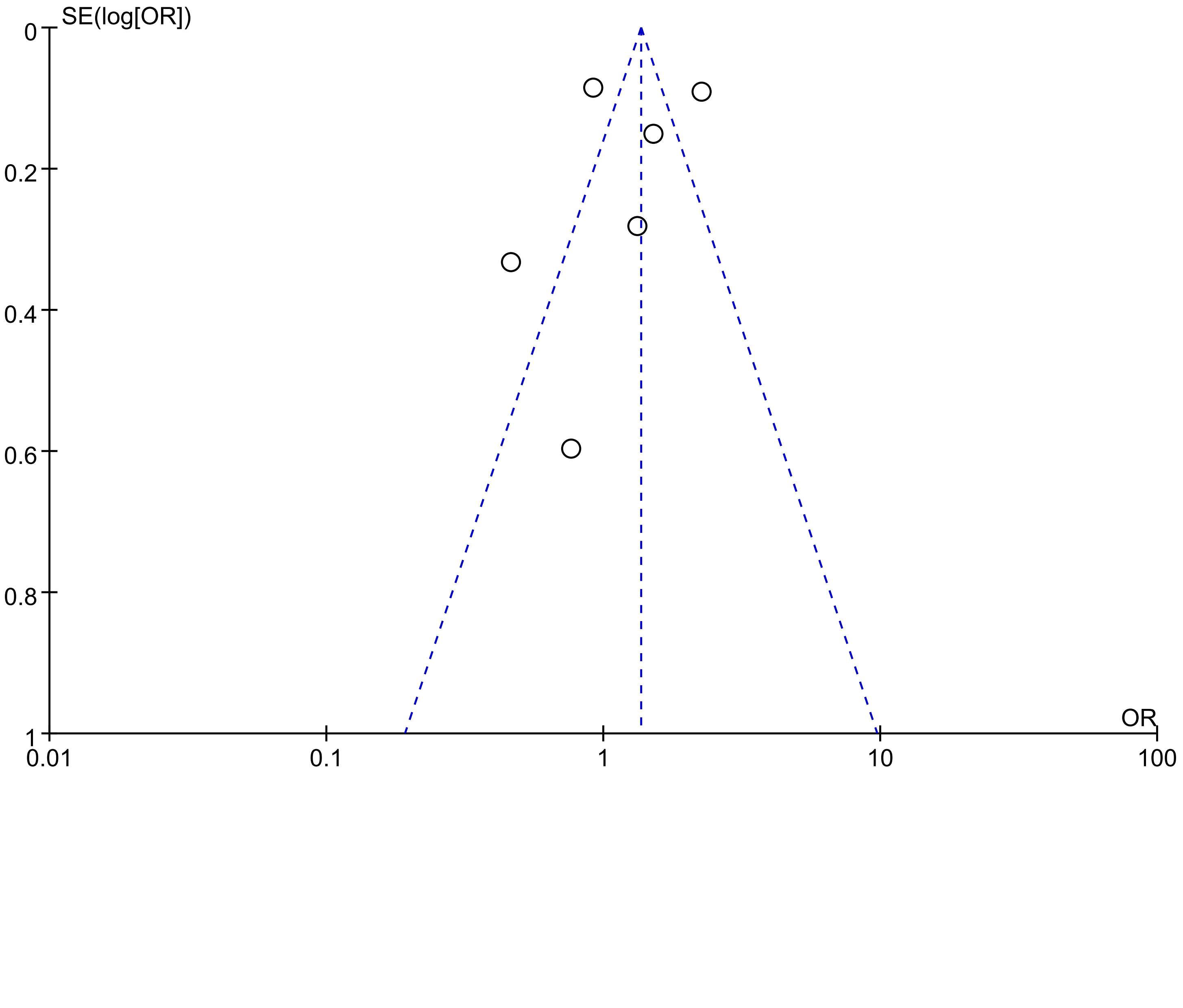

Supplemental Fig. 7

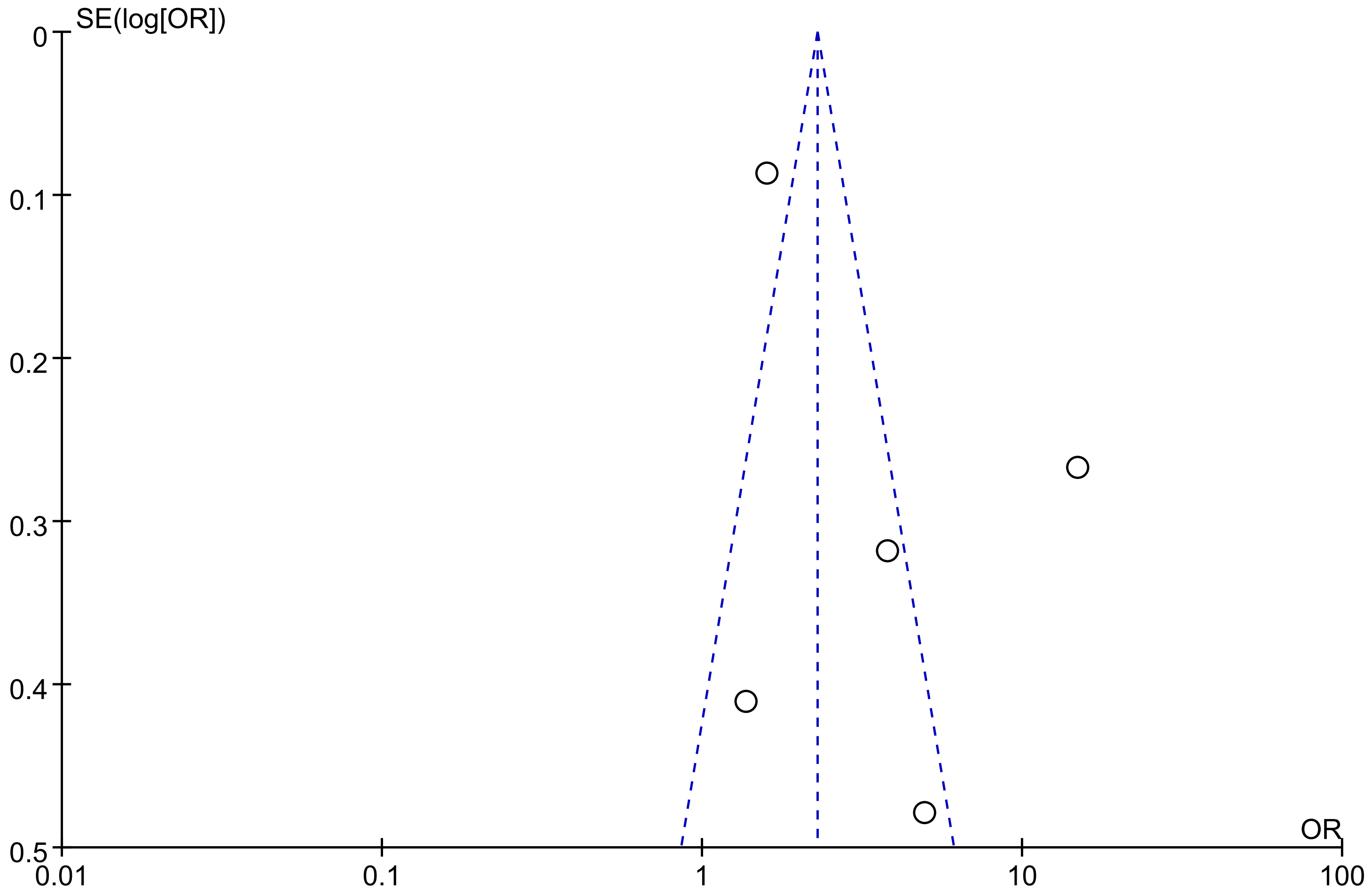

Supplemental Fig. 8A

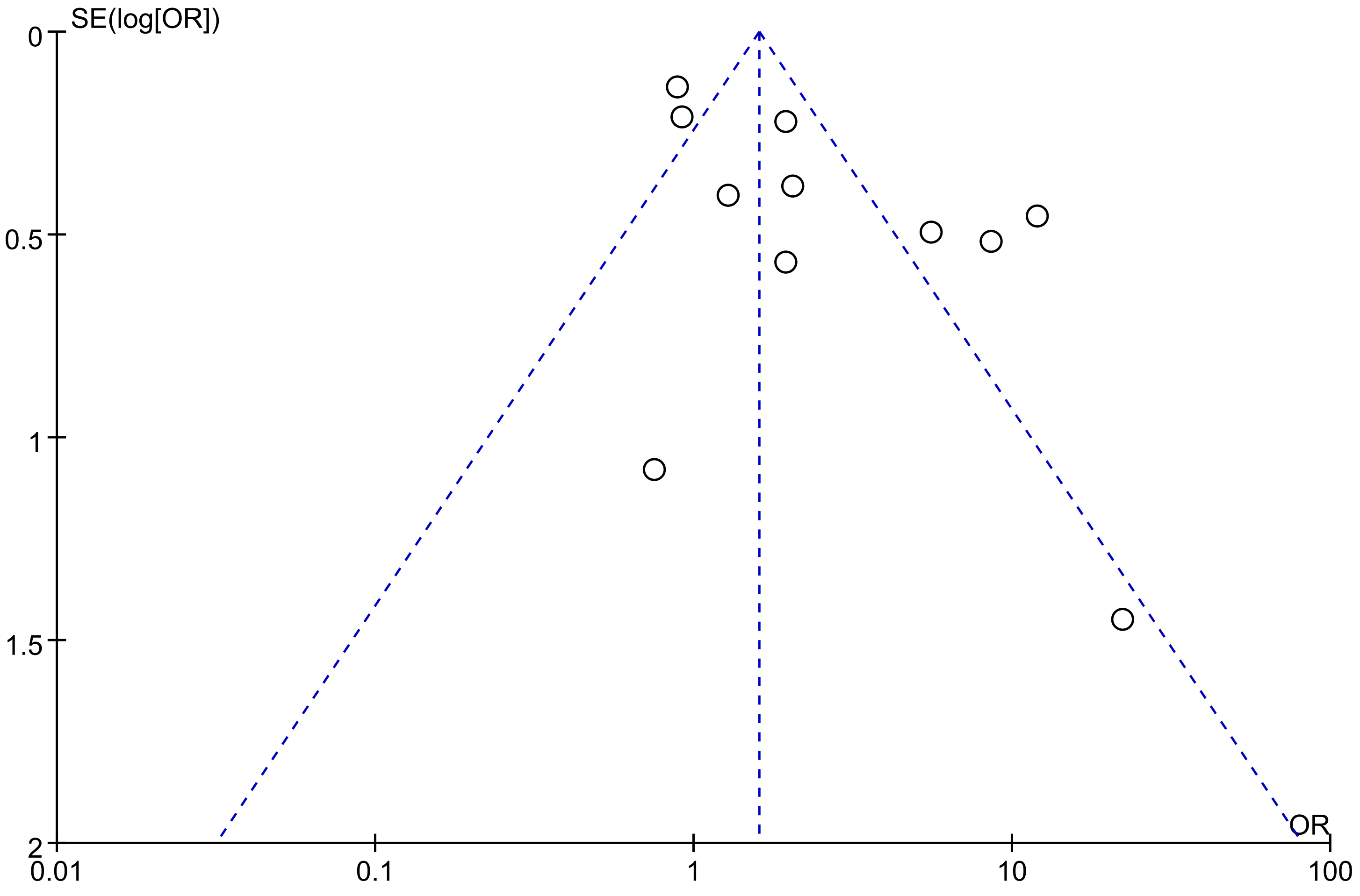

Supplemental Fig. 8B

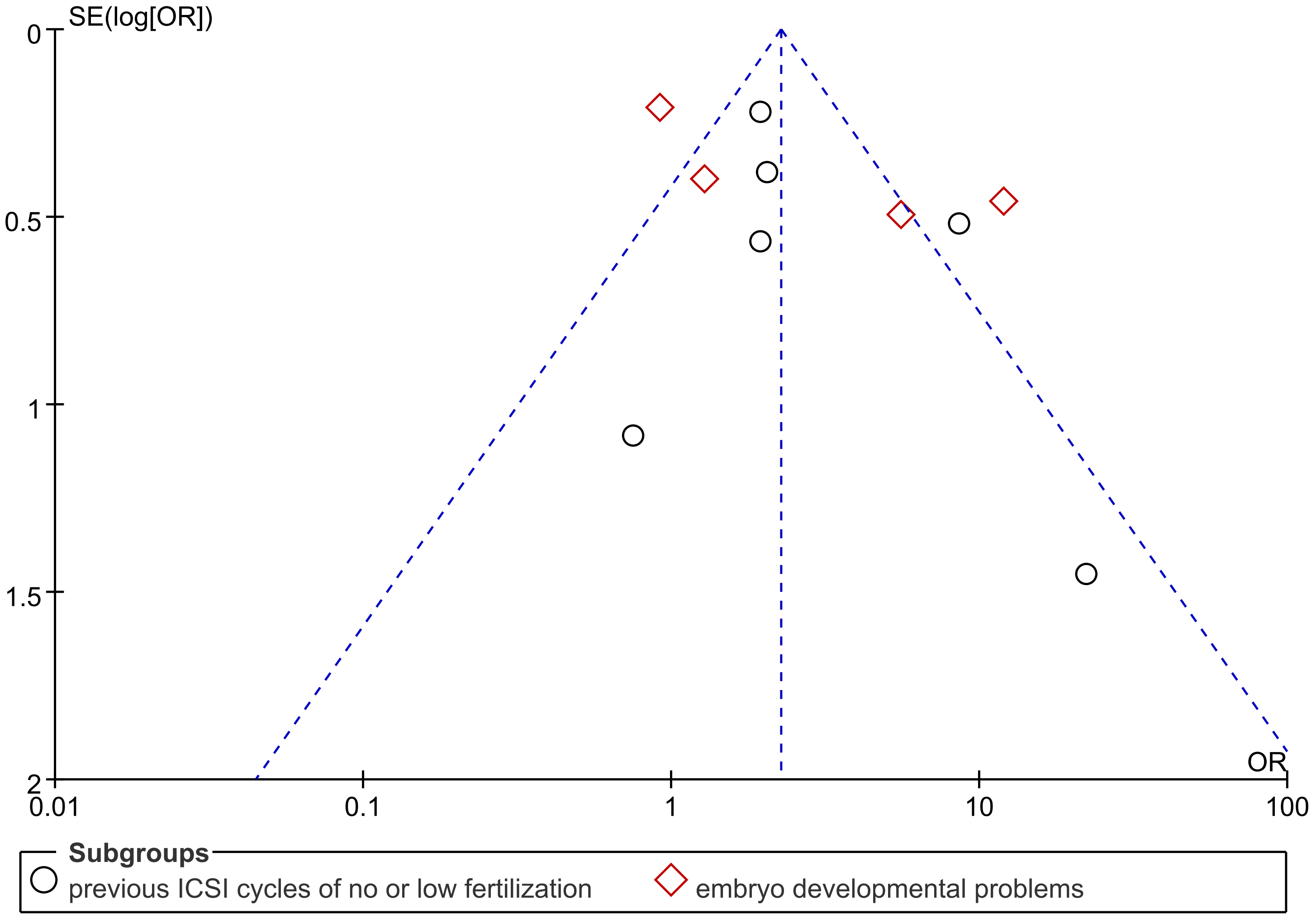

Supplemental Fig. 9

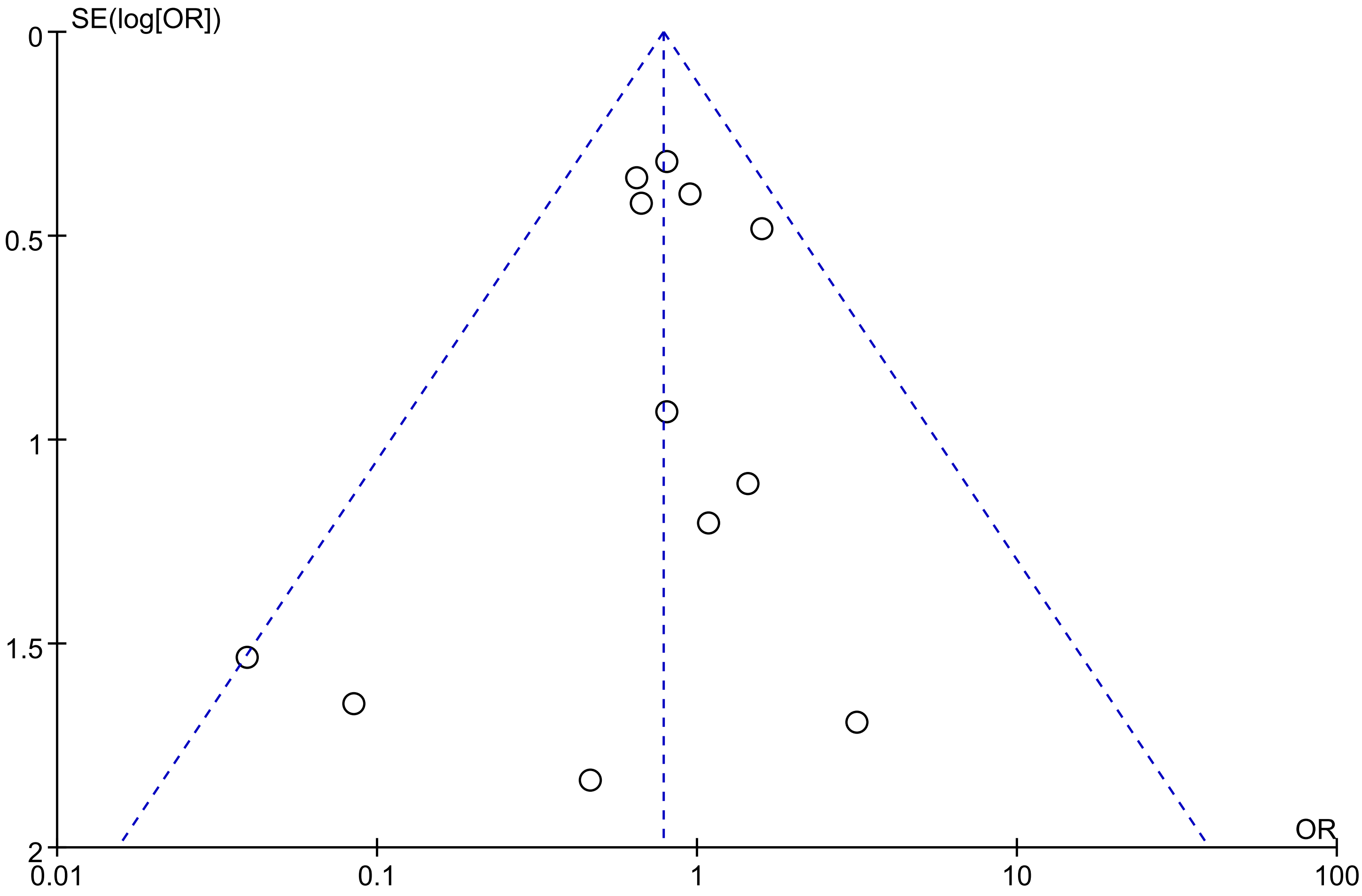

Supplemental Fig. 10A

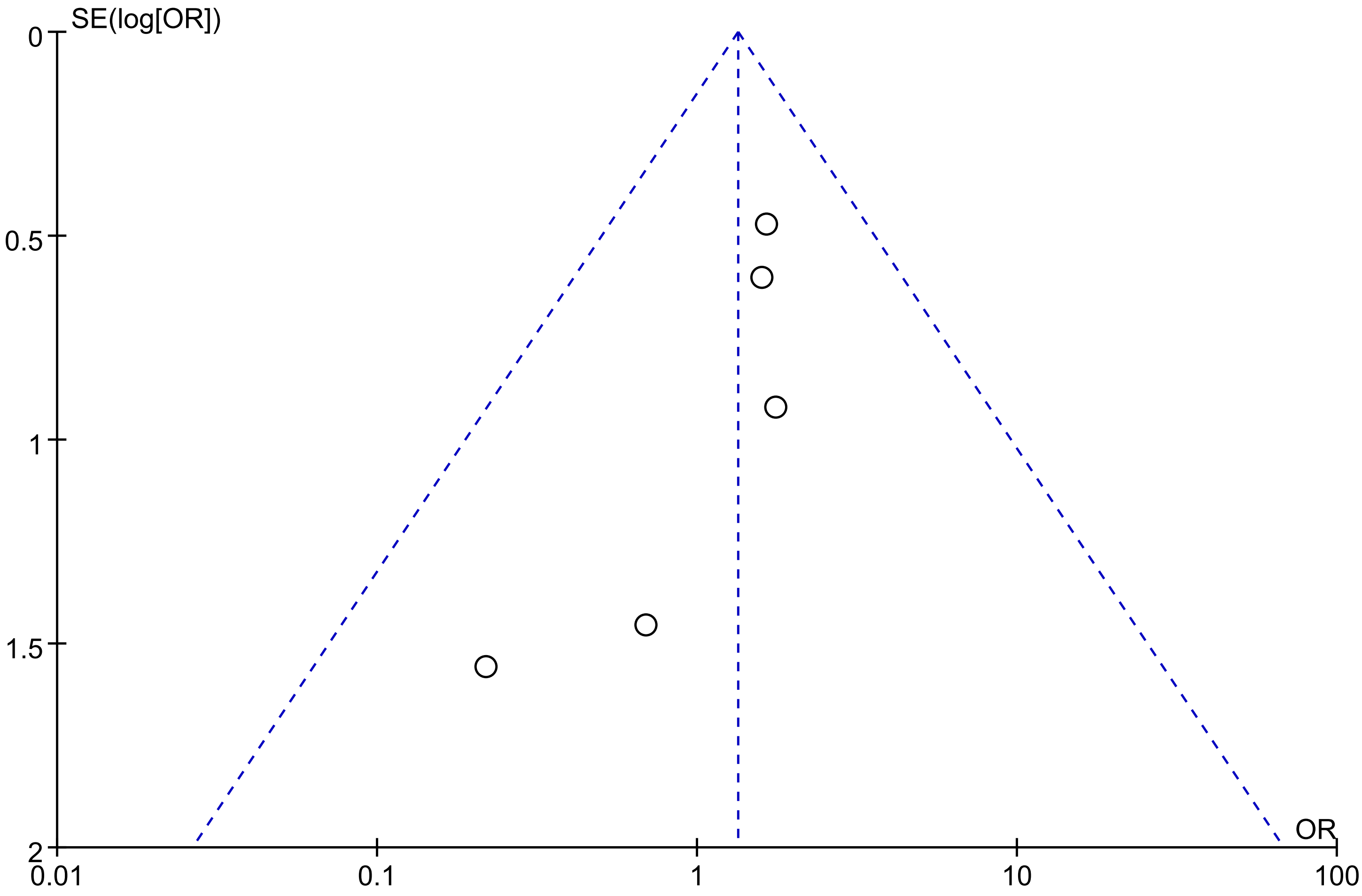

Supplemental Fig. 10B

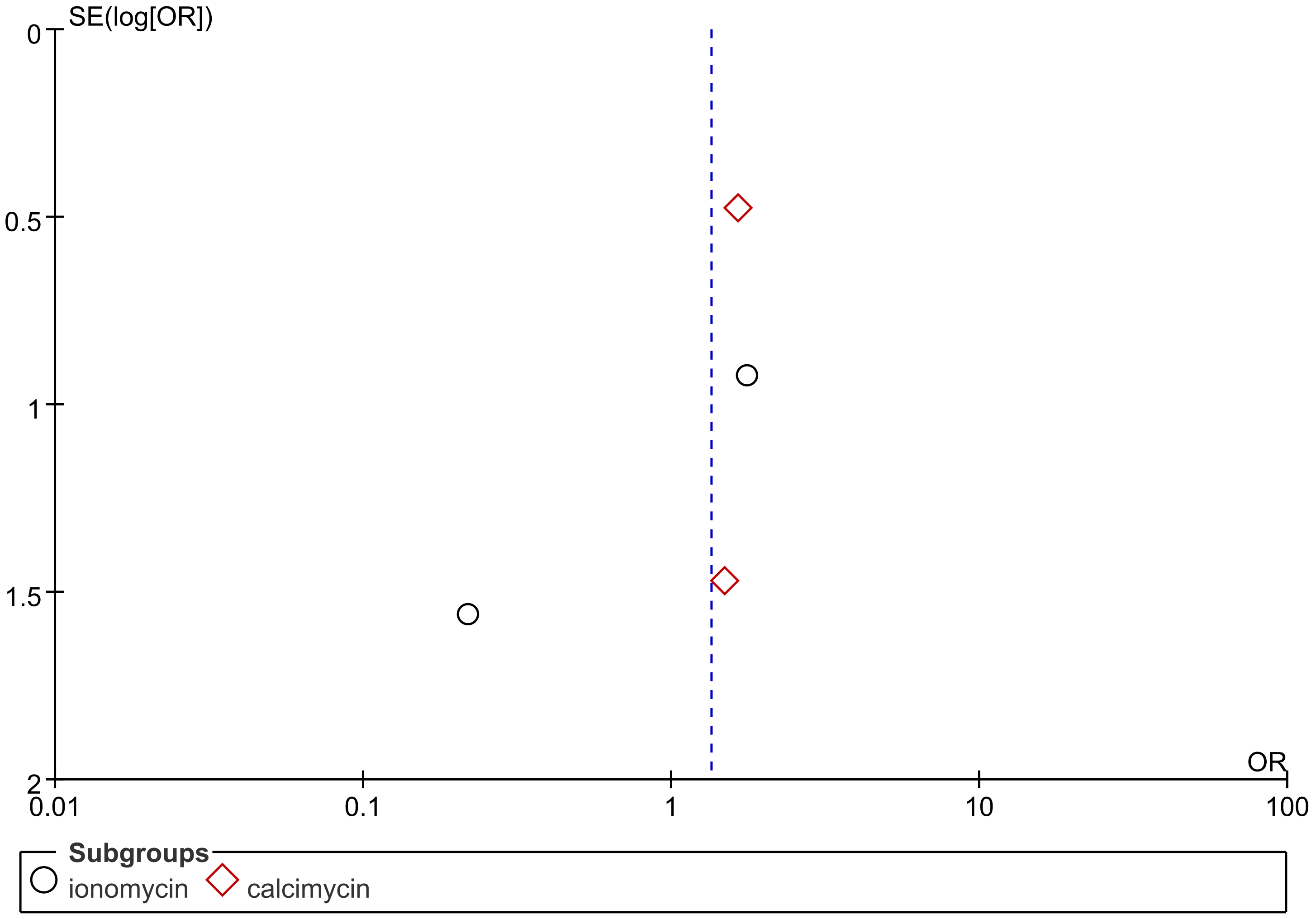

Supplemental Fig. 11

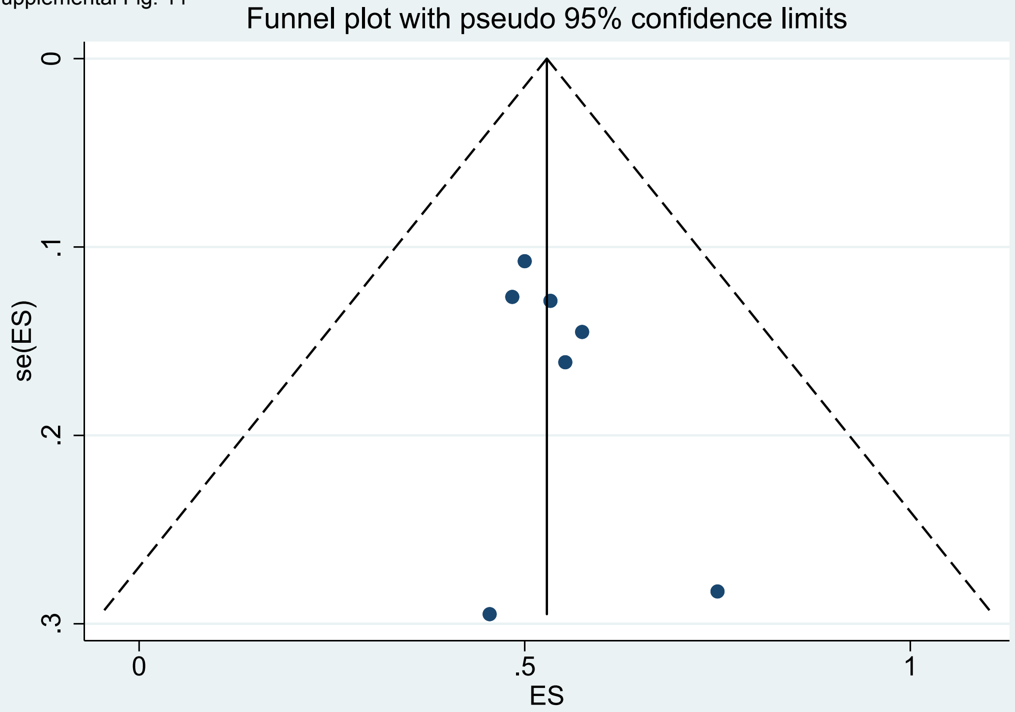

Supplement: Supplementary Figure 1 — Risk of bias of the included RCTs. [file Data_Sheet_1.pdf]
